# Combination of Electroacupuncture and Medication for Patients With Tinnitus Associated With Idiopathic Sudden Sensorineural Hearing Loss: Protocol for a Prospective Randomized Controlled Trial

**DOI:** 10.2196/69163

**Published:** 2025-05-14

**Authors:** Tuoran Wang, Lingyu Qi, Xinping Hao, Miao Zhang, Shihao Du, Biao Chen, Chang Liu, Jing Chen, Xinyue Zou, Ping Liu, Jin Huo

**Affiliations:** 1 Institute of Acupuncture and Moxibustion China Academy of Chinese Medical Sciences Beijing China; 2 Department of Otorhinolaryngology, Head and Neck Surgery Beijing Tongren Hospital Capital Medical University Beijing China

**Keywords:** idiopathic sudden sensorineural hearing loss, tinnitus, acupuncture, study protocol, electroacupuncture, adjunctive therapy

## Abstract

**Background:**

Tinnitus, a common and troublesome symptom of idiopathic sudden sensorineural hearing loss (ISSNHL), is the perception of sound without any external or internal sound source. The etiology of ISSNHL accompanied by tinnitus remains unclear. Tinnitus often occurs alongside or before hearing loss and can severely impact patients’ quality of life, leading to psychological stress and emotional distress. Current treatments primarily focus on improving hearing loss while neglecting tinnitus treatment. Electroacupuncture (EA), a technique widely applied in China for tinnitus management, may become a promising intervention by addressing both auditory and psychological symptoms.

**Objective:**

This study aims to conduct a double-center, assessor-blinded, prospective randomized pilot clinical trial to explore the potential effects and feasibility of EA combined with medication for patients with ISSNHL accompanied by tinnitus, and to generate preliminary data to inform future large-scale trials of EA as an adjunctive therapy for this condition.

**Methods:**

In this double-center, assessor-blinded, randomized controlled trial, 60 patients with ISSNHL accompanied by tinnitus will be randomly allocated to either the EA group, receiving EA combined with medication, or the control group, receiving standard medication treatment, for a total treatment duration of 4 weeks. Follow-up assessments will be conducted at weeks 2 and 4, and at 1, 2, and 3 months after treatment. The feasibility primary outcomes are recruitment rate, adherence rate, data completeness, and safety outcomes. The primary outcome measures will include pure tone audiometry, tinnitus matching, and the Tinnitus Handicap Inventory, while the secondary outcome measures will comprise speech-recognition thresholds in noise, the Self-Rating Anxiety Scale, and the 36-item Short Form Health Survey Questionnaire.

**Results:**

This study began recruitment on July 15, 2024, and is scheduled to conclude on August 31, 2025. To date, 42 participants have been enrolled, with 35 having completed the intervention and posttreatment assessments (control group: n=21; EA group: n=14). Following protocol publication, data compilation and analysis will be conducted, with results anticipated to be published in a relevant journal in 2026.

**Conclusions:**

This pilot trial is expected to provide critical insights into the feasibility of integrating EA with standard medication for managing ISSNHL with tinnitus. By generating preliminary evidence on its practicality and potential benefits, this study aims to inform the design and sample size estimation of future multicenter trials, potentially advancing nonpharmacological treatment options for this challenging condition.

**Trial Registration:**

Chinese Clinical Trial Registry ChiCTR2400086802; https://tinyurl.com/mtxthpzj

**International Registered Report Identifier (IRRID):**

DERR1-10.2196/69163

## Introduction

Idiopathic sudden sensorineural hearing loss (ISSNHL) is characterized by a rapid, unexplained sensorineural hearing loss of at least 30 dB over 3 contiguous frequencies within 72 hours, typically occurring unilaterally [1]. The incidence of ISSNHL is estimated to range from 5 to 20 cases per 100,000 individuals annually. However, some studies suggest that this figure may be underestimated, as those who recover swiftly often do not seek medical care [2]. The tinnitus associated with ISSNHL lacks a precise definition. Clinically, it is typically described as tinnitus that arises simultaneously with, or within hours to days after, the onset of ISSNHL. In some cases, tinnitus may even precede the hearing loss by a few hours to several days. However, patients often pay more attention to symptoms such as tinnitus, aural fullness, or dizziness, which may lead them to overlook the initial onset of ISSNHL. Previous clinical studies have reported a tinnitus incidence ranging from 69.8% to 93.3%. A multicenter study in China found that, among 1024 patients with ISSNHL, 90.1% experienced tinnitus—a prevalence notably higher than that of vestibular symptoms, aural fullness, or dizziness [3,4]. Patients with tinnitus frequently endure considerable psychological stress, mood disturbances, and sleep disorders, all of which can impair their performance at work or in their studies. In more severe cases, these individuals may also develop anxiety and depression [2,5]. The alleviation of tinnitus symptoms typically progresses more slowly than the recovery of hearing. Even after hearing is restored, some patients may continue to experience persistent tinnitus, which can significantly diminish their quality of life. Consequently, clinicians should prioritize addressing tinnitus in tandem with treating hearing loss [6,7].

The pathogenesis of ISSNHL with tinnitus remains unclear [8]. It is generally believed that the onset and progression of tinnitus are related not only to peripheral hearing impairment but also to abnormal brain activity [9], including disrupted intra- and internetwork connectivity, psychological factors, and stress hormones [10,11]. Currently, treatment for ISSNHL accompanied by tinnitus primarily aims to address the underlying hearing loss [6,12]. Restoring auditory function can help disrupt the mechanisms that induce tinnitus, reestablish central auditory input, and prevent its progression [13,14]. The standard treatment approach involves corticosteroid administration, either systemically or through intratympanic injection [1]. The American clinical practice guideline for ISSNHL [1] does not provide specific recommendations for the treatment of comorbid tinnitus, but it highlights its prevalence, refractoriness, and the potential economic and psychological burdens associated with the condition.

The Chinese guideline for the diagnosis and treatment of sudden hearing loss [15] classifies ISSNHL into 4 types based on audiometric patterns: low-frequency descending, high-frequency descending, flat, and total. Different audiometric patterns may suggest distinct pathogenic mechanisms for ISSNHL. For example, the low-frequency descending type is associated with endolymphatic hydrops, the high-frequency descending type with hair cell damage, and the flat type may reflect a broader inner ear injury mechanism, such as stria vascularis dysfunction or inner ear vascular spasm [16]. These differences may influence treatment responses and prognosis [17].

Acupuncture, a therapy that involves the insertion of fine needles into the body followed by manipulation techniques such as lifting, thrusting, and twisting, was described as a treatment for tinnitus as early as the 5th century BCE in China [18]. Although the clinical practice guideline developed by Tunkel et al [19] does not mention acupuncture for the treatment of persistent tinnitus due to the lack of high-quality randomized controlled trials (RCTs), the expert panel acknowledged that many patients with persistent or bothersome tinnitus seek acupuncture therapy. Additionally, a European epidemiological study showed that the average annual out-of-pocket expenditure for European patients with tinnitus was €565 (US $640), with €183 (US $207) spent on acupuncture, homeopathy, and osteopathy. These patients were willing to pay 1.6-7 times their monthly income for complete tinnitus relief [20]. Furthermore, emerging evidence suggests that acupuncture, including electroacupuncture (EA), may modulate functional connectivity within brain networks, such as those involving the insula and frontal regions, potentially contributing to its therapeutic effects on tinnitus and possibly extending to cases associated with ISSNHL [21-24]. EA is a safe and effective intervention that is easier to standardize and more controllable than manual acupuncture, providing prolonged stimulation, precise intensity control, and enhanced acupuncture sensation [25,26].

This study aims to investigate the feasibility and potential effects of EA combined with medication on hearing and tinnitus outcomes in patients with ISSNHL and tinnitus, generating preliminary data to support future research on EA as an adjunctive treatment for ISSNHL.

## Methods

### Design and Setting

**Figure 1:**
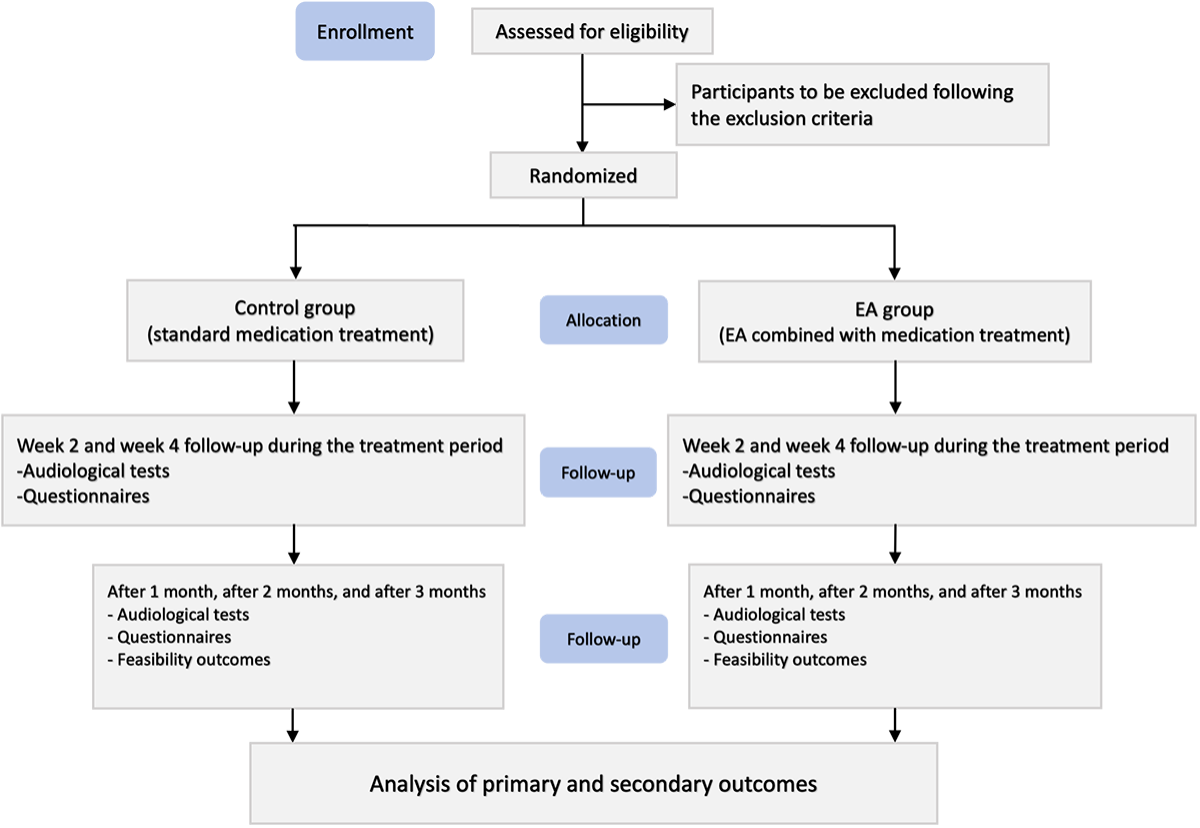
The enrollment process of the trial. EA: electroacupuncture.

The study is designed as a prospective, single-blinded RCT. It will take place in the clinics of Beijing Tongren Hospital, Capital Medical University, and the Acupuncture and Moxibustion Hospital, China Academy of Chinese Medical Sciences (CACMS). Eligible participants will be randomized in a 1:1 ratio to either the EA group (EA plus medication) or the control group (standard medication alone). This pilot trial will apply a longitudinal follow-up design, with a baseline assessment before enrollment (t–1), allocation and intervention initiation at t0, on-treatment follow-ups at week 2 (t1), week 4 (t2), and posttreatment follow-ups at 1 month (t3), 2 months (t4), and 3 months (t5) after intervention completion, barring loss to follow-up. This trial will be reported in accordance with the CONSORT (Consolidated Standards of Reporting Trials)-EHEALTH (see Multimedia Appendix 1) statement, which defines standard reporting items for RCTs [27]. Participant recruitment began on July 15, 2024, with the study scheduled to continue until December 2025. The flowchart is illustrated in Figure 1, and the overall procedure is presented in Table 1.

**Table 1:** Overall procedure of this study.

Stages					Enrollment		Allocation		On-treatment follow-up				Posttreatment follow-up						
Time point					–t_1_		t_0_		t_1_		t_2_		t_3_		t_4_		t_5_		
**Enrollment**																			
	Eligibility screening			✓		N/A^a^		N/A		N/A		N/A		N/A		N/A			
	Informed consent			✓		N/A		N/A		N/A		N/A		N/A		N/A			
	Allocation			N/A		✓		N/A		N/A		N/A		N/A		N/A			
**Interventions**																			
	Control group			N/A		N/A		✓		✓		N/A		N/A		N/A			
	Electroacupuncture group			N/A		N/A		✓		✓		N/A		N/A		N/A			
**Assessments**																			
	**Feasibility outcomes**																		
		Recruitment rate		N/A		N/A		N/A		N/A		✓		N/A		N/A			
		Adherence rate		N/A		N/A		N/A		N/A		✓		N/A		N/A			
		Data completeness		N/A		N/A		N/A		N/A		✓		N/A		N/A			
		Adverse events		N/A		N/A		✓		✓		✓		✓		✓			
	**Exploratory outcomes**																		
			Pure tone audiometry	N/A		✓		✓		✓		✓		✓		✓			
			Tinnitus matching	N/A		✓		✓		✓		✓		✓		✓			
			Tinnitus Handicap Inventory	N/A		✓		✓		✓		✓		✓		✓			
	**Secondary outcomes**																		
			Speech-recognition thresholds in noise	N/A		✓		✓		✓		✓		✓		✓			
			Self-Rating Anxiety Scale	N/A		✓		✓		✓		✓		✓		✓			
			36-item Short Form Health Survey Questionnaire	N/A		✓		✓		✓		✓		✓		✓			

^a^N/A: not applicable.

### Eligibility Criteria

Inclusion criteria included the following: a diagnosis of ISSNHL [15], defined as sudden hearing loss (≥30 dB) within 72 hours affecting 3 consecutive frequencies, and accompanied by tinnitus; between 18 and 65 years of age at the time of recruitment; pure tone audiometry (PTA) results showing a flat drop audiogram; onset of hearing loss within 1 week before randomization; clear consciousness, normal cognition, and unaffected basic communication.

Exclusion criteria were hearing loss from other causes (eg, congenital deafness, conductive deafness, presbycusis, large vestibular aqueduct syndrome, Meniere disease, tumors, or deafness due to systemic genetic diseases); having received medication or acupuncture treatment within the past 6 months, or having an allergy to the treatment medication; pregnancy or lactation in females; presence of severe systemic diseases or psychiatric disorders; and participation in other trials.

### Ethical Considerations

Ethics approval was obtained from the Ethics Committee of Beijing Tongren Hospital, Capital Medical University (approval number TREC2024-KY060). Before the trial begins, researchers must provide participants with detailed information about the clinical trial, including its purpose, procedures, potential benefits and risks, alternative treatment options, and the rights and responsibilities of the participants. Participants will also be informed that they are free to withdraw consent at any time during the study. They must fully understand this information and provide their consent by signing an “informed consent form” before the clinical trial can commence.

### Enrollment, Randomization, and Blinding

Recruitment will be conducted through WeChat (Tencent Holdings Limited) public account notifications, roll-up banners at clinic entrances, and collaboration with community committees for promotion.

Stratified block randomization will be performed according to the study center. Both groups will be assigned in a 1:1 ratio to the EA and control groups using a block size of 4. Weijuan Gang, a statistician from the Evidence-Based Medicine Center at CACMS, will serve as the randomization coordinator and will be responsible for generating the stratified block randomization sequence using SAS 9.3 software (SAS Institute). She will not participate in the trial’s implementation or data analysis. After obtaining informed consent and before initiating treatment, the treating physician at each center will contact the randomization coordinator by phone to receive the randomization number and group assignment.

Because of the nature of acupuncture, it is not possible to blind the acupuncturists or participants. However, steps will be taken to minimize bias from single blinding as much as possible. Acupuncturists will receive specialized training before participating in the study and will be instructed not to discuss treatment procedures or outcomes. They will also be excluded from evaluating the results. Patients will be treated separately to prevent contact between participants. The randomization staff and acupuncturists will have access to the assignment information, but the outcome evaluators and statisticians will remain blinded throughout the entire clinical trial. Statisticians will speculate on the group allocation before data analysis to assess the efficacy of blinding, and unblinding will only occur after the completion of data analysis.

### Sample Size

As previous RCTs have not investigated EA as a combination intervention in patients with ISSNHL with tinnitus, it is challenging to determine the optimal sample size through power calculation. Therefore, rather than performing a formal sample size estimation, this trial will adopt a preliminary exploratory clinical pilot design to estimate the appropriate sample size [28]. However, to preliminarily assess the feasibility of the proposed intervention, we will ensure an adequate number of participants in our experimental design by adhering to the established guideline of a minimum of 12 participants per group for pilot studies [29]. A previous study suggested that each group should have a sample size ranging from 20 to 25 cases for exploratory clinical trials assessing the effectiveness of intervention measures [30]. Therefore, in this study, each group will consist of 25 patients, resulting in a total of 50 cases across the 2 groups. To account for a potential dropout rate of 20%, we will recruit a total of 60 patients (30 per group). The results of this study will provide effect size data for calculating sample sizes in future large-scale RCTs.

### Interventions

The control group intervention protocol was designed in accordance with the 2015 Chinese Guidelines for the Diagnosis and Treatment of Sudden Deafness [15]. These guidelines were developed based on extensive RCT data from multicenter clinical studies on sudden deafness in China, incorporating updates from the latest American and German clinical guidelines as well as recent research advancements, ensuring alignment with clinical practice in China [1,4,16]. The treatment regimen for the control group was designed to comprehensively address the inflammation and microcirculatory disturbances associated with ISSNHL through a combination of prednisone, Ginkgo biloba extract, and batroxobin injection. The specific pharmacological interventions are detailed in Table 2.

**Table 2:** Standard treatment regimen for the control group.

Period	Drug	Dosage and administration	Duration
Weeks 1-2 after onset	Prednisone (glucocorticoid)	1 mg/kg/day (maximum 60 mg), oral, once daily in the morning	5 days
Weeks 1-2 after onset	Ginkgo biloba extract	87.5 mg in 250 mL 5% glucose, intravenous infusion, once daily	2 weeks
Weeks 1-2 after onset	Batroxobin injection	5 batroxobin units in 250 mL 5% glucose, intravenous infusion, every other day^a^	2 weeks
Weeks 3-4 after onset	Ginkgo biloba tablets	9.6 mg × 2 tablets, oral, 3 times daily	2 weeks

^a^Before the administration of batroxobin, blood tests are required to confirm fibrinogen levels (>1.5 g/L) and normal coagulation parameters (eg, prothrombin time or activated partial thromboplastin time). Fibrinogen and coagulation function are monitored 24 hours after the first dose and periodically thereafter.

Patients in the EA group will receive the same treatment as the control group, in addition to EA, which will not interfere with their medication treatment.

**Figure 2:**
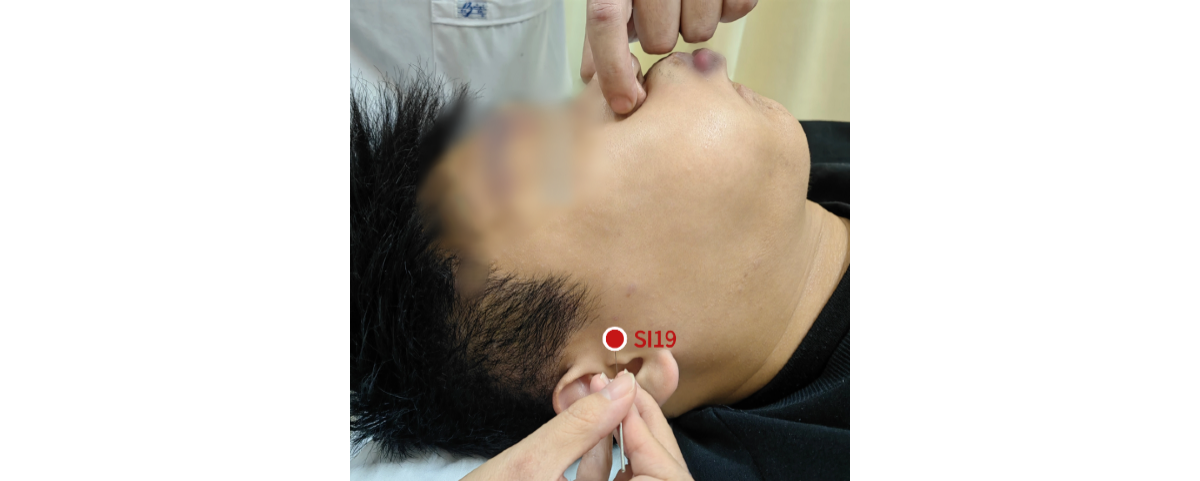
The operational illustration of acupuncture technique at SI19.

**Figure 3:**
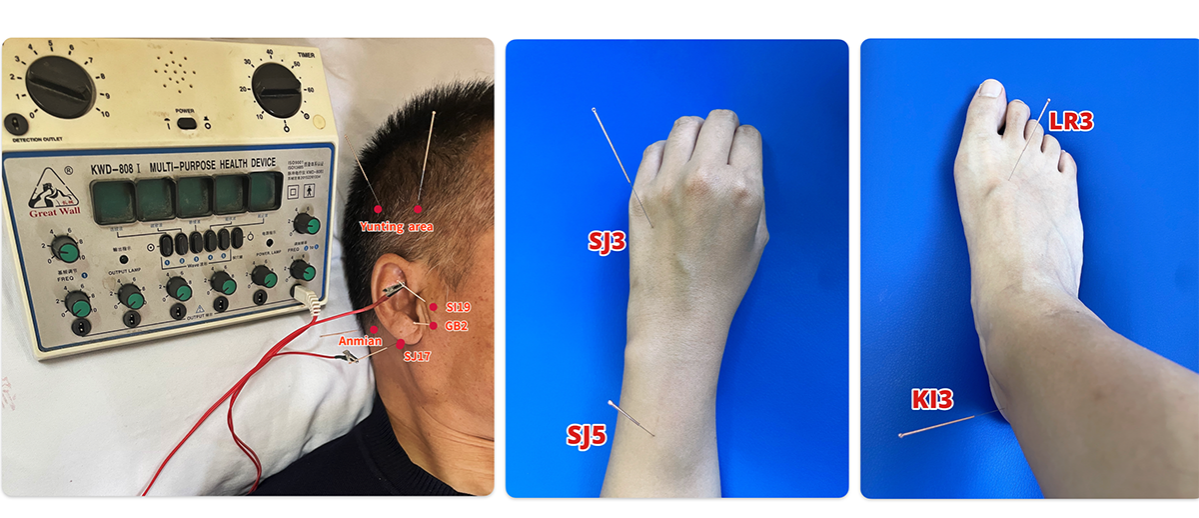
The acupuncture point selection diagram.

The EA group received acupuncture using disposable sterile needles (Hwato Needles; Suzhou Medical Appliance Factory) at acupoints previously identified in studies as functionally related to ISSNHL and tinnitus, including Tinggong (SI19), Tinghui (GB2), Yifeng (SJ17), Yunting area, Anmian, Waiguan (SJ5), Zhongzhu (SJ3), Taichong (LR3), and Taixi (KI3). For limb acupoints, 30-gauge needles (0.25 mm in diameter, 40 mm long) will be inserted to a depth of 10-15 mm. For head acupoints, 32-gauge needles (0.25 mm in diameter, 25 mm long) will be inserted to a depth of 3 mm. A specialized acupuncture technique will be used when needling the Tinggong point (SI19) on the impacted side. The patient will be instructed to slightly open their mouth, and a needle (0.25 mm in diameter, 40 mm long) will be inserted to a depth of approximately 15-25 mm. The assistant will pinch the patient’s nostrils, prompting the patient to take a deep breath, close their mouth, and hold their breath to push air through the Eustachian tube into both ears. During this procedure, the acupuncturist will gently twist the needle until the patient feels a local sensation of soreness or distension. Afterward, the patient will exhale through the mouth, and the acupuncturist will withdraw the needle by approximately 10 mm and leave it in place. Additionally, the Tinghui (GB2) and Yifeng (SJ17) points on the impacted side will be needled and connected to an EA device (KWD-808I type; Great Wall, Yingdi). A sparse wave with a frequency of 2-15 Hz will be applied at an intensity that induces muscle contraction and is tolerable for the patient. The needles will be retained for 30 minutes. Figure 2 provides an operational illustration of the acupuncture technique at the Tinggong (SI19) point, while Figure 3 shows the acupuncture point selection diagram. EA treatment will last for a total of 4 weeks, with 4 sessions per week during the first 2 weeks and 3 sessions per week during the last 2 weeks, conducted every other day.

### Outcome Measures

#### Feasibility Primary Outcomes

The feasibility primary outcomes are recruitment rate, defined as the proportion of enrolled participants relative to the target sample size; adherence rate, measured as the proportion of participants completing at least 80% of the prescribed EA sessions over 4 weeks, as extracted from treatment logs; data completeness, assessed as the proportion of participants with fully collected baseline and follow-up data for key measures such as PTA and Tinnitus Handicap Inventory (THI); and safety outcomes, recorded as the incidence of adverse events during the treatment period, based on session-by-session reports [31,32].

#### Exploratory Primary Outcomes

##### Overview

PTA threshold tests will be performed using an audiometer (Otometrics Conera, Natus Medical Incorporated/GN Otometrics A/S) in a standard soundproof room with background noise less than 30 dB(A) to minimize the influence of human factors on the test results. The patient will be presented with sounds at frequencies ranging from 0.25 to 8 kHz [33,34]. An audiologist will use the audiometer to measure both air conduction and bone conduction hearing thresholds. A shift of more than 30 dB between adjacent frequencies indicates hearing loss. Following these measurements, the audiogram configuration is obtained simultaneously. Hearing loss across all frequencies, represented by a horizontal line with threshold differences of less than 20 dB between frequencies, is classified as a “flat” audiogram type [35].

Tinnitus matching is currently the most objective means of detecting tinnitus. It mainly includes 2 parts [36], as described in Textbox 1.

**Parts of tinnitus matching.**

- **Tinnitus frequency matching**

Tinnitus frequency matching is performed using an audiometer (Otometrics Conera). A pure tone with an intensity similar to the tinnitus sound is presented to the ear opposite to the tinnitus-impacted ear or the more severe side, with the frequency adjusted between 0.25 and 8 kHz. This adjustment continues until the presented pure tone frequency matches or closely resembles the patient’s perceived tinnitus frequency, which is then defined as the tinnitus pitch. For patients who cannot match a pure tone, a narrowband noise frequency is used, and the center frequency of this narrowband noise is defined as the tinnitus pitch. Tinnitus frequencies are categorized as low (<1 kHz), medium (1-4 kHz), and high (≥4 kHz).

- **Tinnitus loudness matching**

After determining the tinnitus pitch, the audiologist matches the loudness at this frequency. Starting from the hearing threshold at the tinnitus pitch, the intensity of the test tone is gradually increased in 1-dB increments until it just masks the tinnitus sound. The difference between the measured sound intensity and the corresponding hearing threshold at this frequency is recorded as the tinnitus loudness [37]. Tinnitus loudness is classified into 5 levels based on the World Health Organization hearing loss grading: ≤25 dB, 26-40 dB, 41-60 dB, 61-80 dB, and ≥81 dB [38].

##### Tinnitus Handicap Inventory

This scale consists of 25 questions and assesses the severity of tinnitus from various aspects, including psychological, social, occupational, physiological functions, and emotional issues such as anger [39]. Responses are scored as follows: “Yes”=4 points, “Sometimes”=2 points, and “No”=0 points, with a maximum possible score of 100 points. Based on the THI score, tinnitus is classified into 5 levels: 1-16 points as grade I (mild tinnitus), 18-36 points as grade II (mild tinnitus), 38-56 points as grade III (moderate tinnitus), 58-76 points as grade IV (severe tinnitus), and 78-100 points as grade V (catastrophic tinnitus) [40]. In this proposed study, the validated Chinese version of the THI, which has high test-retest reliability (*r*=0.98) and internal consistency (Cronbach α=0.93), will be used [41].

#### Secondary Outcomes

##### Speech-Recognition Thresholds in Noise

Speech audiometry in background noise effectively simulates real-life conditions, providing a more accurate representation of a participant’s hearing ability in everyday social interactions [42]. The test will be conducted using the HOPE automated software (ADDI Medical AB), following the speech-recognition thresholds in noise (SRT) testing guidelines recommended by the American Speech-Language-Hearing Association. The SRT calculation will be as follows: SRT = initial test level – number of correctly repeated words + 5 dB (correction factor) [43].

##### Self-Rating Anxiety Scale

The Self-Rating Anxiety Scale (SAS) [44] consists of 20 items and uses a self-report format to assess anxiety symptoms that may have occurred in the past week, covering emotional, physical, and behavioral aspects. After calculating the original total score, it is multiplied by 1.25 to obtain a standard score (range 25-100). Higher scores indicate more severe anxiety symptoms.

##### 36-Item Short Form Health Survey Questionnaire

The 36-Item Short Form Survey Questionnaire (SF-36) [45] is a self-reported questionnaire that assesses health-related quality of life over the past 2 weeks to 1 month across 36 items, covering 8 health dimensions. For each dimension, the final score is calculated by converting the raw score using the formula: final score = (actual score – minimum possible score)/(maximum possible score – minimum possible score) × 100. Each dimension is scored on a scale from 0 to 100, with higher scores indicating better quality of life in that domain.

### Program Safety and Feasibility

#### Study Oversight and Standardization of Clinical Procedures

The trial protocol has undergone rigorous review by a multidisciplinary team, including acupuncturists, otolaryngologists, audiologists, and statistical experts. Investigators will be selected following a qualification assessment and will remain consistent throughout the study. All team members—including acupuncturists, otolaryngologists, and audiologists—will receive standardized prestudy training to ensure uniform implementation of the protocol. To monitor study progress, address emerging issues, and ensure protocol adherence, regular meetings will be held twice a month. Hearing assessments will be conducted in accordance with standardized operating procedures. Any sudden changes in hearing or adverse reactions to acupuncture during the study will be promptly managed. All acupuncture treatments will be administered exclusively by practitioners with over 2 years of clinical experience, and no other techniques, such as acupressure or intradermal needle application, will be used. Senior physicians will supervise the entire process, providing guidance and resolving any issues to ensure the smooth and consistent delivery of treatment.

#### Management of Adverse Drug Reactions

If a drug-related allergic reaction occurs, the offending medication will be immediately discontinued. Patients will be advised to increase fluid intake to facilitate drug excretion. Antihistamines such as loratadine or cetirizine will be administered as needed, and calamine lotion may be applied topically to relieve localized symptoms.

#### Management of Acupuncture-Related Adverse Events

##### Needle Fainting

In the event of needle fainting, all acupuncture needles will be promptly removed. The patient will be assisted into a supine position in a well-ventilated area, with the legs elevated and the head positioned lower, without a pillow. The patient will be allowed to rest quietly and will be offered warm sugar water or hot tea to aid recovery.

##### Stuck Needle

If a needle becomes stuck, especially in anxious patients, the retention time may be extended while gently massaging the area around the acupuncture point to promote relaxation. If the needle remains immobile, a secondary needle may be inserted near the original point to help release tension. In cases where the needle is stuck due to unidirectional rotation, it should be twisted gently in the opposite direction. Additional techniques, such as scraping or flicking the needle handle, may be applied to release entrapped muscle fibers and facilitate safe needle removal. For bleeding around the acupuncture site, a cold compress or pressure will be applied as needed. Participants may choose to withdraw from the intervention or study at any time and for any reason. They will be informed about the permission, consent, and assent forms and may discontinue participation at any point without any consequences.

Given the disease characteristics of hearing loss and the professional level of the hospitals conducting the study in China, participant compliance is expected to be high. To further support compliance, we will provide regular hearing assessments, offer health education, and provide a compensation of 200 RMB for transportation and time costs.

### Oversight and Monitoring

The principal investigators (JH and XH) will oversee the trial and report any serious adverse events to the Tongren Hospital Ethics Committee. Detailed records of adverse events—including their timing, severity, and treatment—will be maintained. In the event of an adverse reaction, a senior acupuncturist and otolaryngologist will evaluate whether the patient should be excluded from the study, and the clinical supervisor will be informed accordingly. Researchers will document the event details in the case report form (CRF; Multimedia Appendix 2) and ensure follow-up care until the patient stabilizes, with a summary report sent to the relevant authorities. Audiologists, acupuncturists, and nurses will conduct weekly follow-up calls to monitor symptoms and manage potential adverse reactions.

### Data Collection and Management

This RCT will adhere to the principles outlined in the Declaration of Helsinki [46]. Written informed consent will be obtained from all participants, with personal data and consent forms securely stored in a locked unit in the ENT (ear, nose, and throat) outpatient department at Tongren Hospital. Each participant will be assigned a unique ID to maintain anonymity, and all data collected in the computer system will be identified only by this ID number. Documentation files (including ID numbers and participant identifiers) will be stored separately from study information files and consent forms. Only the principal investigators and designated team members will have access to the participant ID list and associated information. Clinical data will be collected and recorded by trained research evaluators in our outpatient clinic, initially entered on CRFs, and then into the EpiData system (EpiData Association). Each CRF will undergo double entry by 2 staff members, followed by a consistency check to generate a validation report. Any discrepancies will be investigated and corrected. All paper records will be stored securely in a controlled room with appropriate temperature, humidity, and fire safety measures. Digital files will be securely stored on a computer or external hard drive to prevent data loss. If issues arise during the study, database variables, particularly primary ones, may undergo full or sampled manual checks and be cross-referenced with the CRFs for accuracy.

### Statistical Analysis

All efficacy analyses will follow the intention-to-treat principle, including all randomized participants. Statistical analyses will be performed using SPSS version 22.0 (IBM Corp). Continuous variables, including PTA, THI, SAS, and SF-36, will be reported as means (SDs) for normally distributed data, or as medians (IQRs) for nonnormally distributed data. Tinnitus matching (frequency and loudness) will be treated as a categorical variable and summarized as proportions or percentages.

Baseline PTA distributions for both groups will be reported as means (SD) or medians (IQRs). Differences in baseline hearing loss severity will be assessed using the 2-tailed (unpaired) independent samples *t* test (for normally distributed data) or the Mann-Whitney *U* test (for nonnormally distributed data). If significant differences are identified, group comparisons will be adjusted for hearing loss severity using analysis of covariance to ensure comparability.

Changes in PTA, THI, SAS, and SF-36 across 6 time points (t0, t1, t2, t3, t4, and t5) will be evaluated using repeated measures analysis of variance for normally distributed data, or the Friedman test for nonnormally distributed data. Changes in tinnitus matching across the 6 time points will be assessed using the Cochran *Q* test to detect overall differences. If significant, pairwise comparisons will be conducted using the McNemar test with a Bonferroni correction for multiple comparisons.

Group differences in PTA, THI, SAS, and SF-36 at each time point will be analyzed using the independent samples *t* test (for normally distributed data) or the Mann-Whitney *U* test (for nonnormally distributed data). If baseline hearing loss severity differs significantly, analysis of covariance will be applied to adjust for this covariate. Differences in tinnitus matching at each time point will be evaluated using the chi-square test or Fisher exact test, as appropriate. Statistical significance will be set at α=.05 (2-sided).

As a pilot study, the primary feasibility outcomes include the proportion of enrolled participants relative to the target sample size (recruitment rate, reported as a percentage), the proportion of participants completing at least 80% of prescribed EA sessions over 4 weeks (adherence rate, extracted from treatment logs and reported as a percentage), the proportion of participants with complete baseline and follow-up data for key measures such as PTA and THI (data completeness, reported as a percentage, with the proportion of missing data reported as a complementary indicator), and the incidence of adverse events during the treatment period (safety outcomes, recorded from session-by-session reports and summarized as total events and incidence rate per 100 person-sessions). These feasibility outcomes will be presented using descriptive statistics, without hypothesis testing.

## Results

This study began recruitment on July 15, 2024, and is scheduled to conclude on August 31, 2025. To date, 42 participants have been enrolled, with 35 having completed the intervention and posttreatment assessments (control group: n=21; EA group: n=14). Following protocol publication, data compilation and analysis will be conducted, with results anticipated to be published in a relevant journal in 2026.

## Discussion

### Overview

The treatment guidelines for ISSNHL have been clearly established. However, the treatment protocol for tinnitus, a common comorbid symptom of ISSNHL, remains uncertain [1,47]. This study aims to investigate the potential efficacy and feasibility of EA combined with medication for patients with ISSNHL accompanied by tinnitus. Currently, only a limited number of studies have focused on the treatment of tinnitus, with therapeutic approaches including sound therapy and psychological therapy [48]. Because of the varied nature of tinnitus symptom maintenance, a single treatment modality may not be suitable for all patients [49]. Acupuncture, as one of the most widely accepted complementary and alternative therapies in China, has been explored in prior research for its potential in managing ISSNHL and tinnitus. For instance, Zhou and Jin [50] conducted a retrospective study comparing EA with traditional acupuncture and found that EA was more effective in reducing hearing thresholds and alleviating tinnitus over a 3-month treatment period. Similarly, Zhang et al [51] performed a systematic review and meta-analysis of 12 RCTs, demonstrating that manual acupuncture or EA combined with medication outperformed medication alone in improving hearing outcomes, with some evidence suggesting that EA alone was superior to medication. Additionally, Chen and Jing [52] reported 2 refractory cases where EA alone (12 sessions over 4 weeks) led to complete recovery of unilateral sensorineural hearing loss. However, these findings are limited by low evidence quality and the rare use of tinnitus-specific outcomes (eg, tinnitus matching or THI) as primary end points.

The selection of acupuncture points is primarily based on the following rationale. A study by Huang et al [53] revealed that, through data mining analysis of 112 studies containing 221 prescriptions, the 4 most frequently used acupuncture points were SI19, GB2, SJ17, and SJ3. The SI19-GB2-SJ17 point combination was identified as the core acupoint cluster according to association rule analysis. These peri-auricular acupoints may alleviate tinnitus by stimulating auditory-related nerves and improving blood circulation in the ear region [24,54]. Additionally, scalp acupuncture may exert its effects through the trigeminal nerve and blood circulation regulation, contributing to the improvement of auditory processing and neural activity [55,56].

Most studies currently agree that the earlier patients with ISSNHL receive treatment, the better their prognosis. The study by Byl [57] indicated that patients who received medical attention within 7 days of onset had an approximately 56% recovery rate, whereas those who presented after 30 days had a recovery rate of only 27% (also see [58]). The study by Diao et al [59] also demonstrated that the number of days from onset to treatment was an independent prognostic factor, with shorter time intervals correlating with better outcomes. Therefore, all patients selected for this study were within 7 days of onset. The medication treatment protocol for ISSNHL with tinnitus is the same as that for ISSNHL. The inclusion of multiple variables (ie, multiple drugs) in the standard treatment is driven by the complex pathophysiology of ISSNHL. Research indicates that inflammation is likely a key contributing factor, and corticosteroids such as prednisone are used to reduce inner ear inflammation and edema, thereby improving hearing outcomes [60]. Ginkgo biloba extract, known for its circulation-enhancing and antioxidant properties, may help restore cochlear microcirculation [61]. Batroxobin, a snake venom–derived enzyme with fibrinolytic activity, may improve inner ear blood flow by reducing blood viscosity [62]. This combination of drugs is designed to simultaneously target distinct pathological mechanisms, thereby enhancing overall therapeutic efficacy. The rationale for this combined approach is supported by evidence from studies conducted in China. A retrospective study involving 342 patients with total deafness-type ISSNHL found that adding batroxobin to a treatment regimen of Ginaton and glucocorticoids significantly improved hearing recovery outcomes and maintained plasma fibrinogen levels within a safe and prognostically favorable range [63]. This improvement was particularly notable in terms of hemorheological parameters and peripheral blood T-cell subset reactivity. Additionally, a 2023 meta-analysis further corroborated the efficacy of Ginkgo biloba extract as an adjuvant therapy, suggesting it may outperform corticosteroid monotherapy [64]. These findings support the decision to incorporate multiple pharmacological agents into the standard treatment protocol, aligning with the goal of addressing the multifaceted nature of ISSNHL.

### Innovations and Limitations

This study innovatively investigates EA for flat-type ISSNHL, integrating traditional Chinese medicine with modern clinical needs to explore a potential nonpharmacological treatment option. However, there are several limitations to this study. First, due to the inability to implement blinding in the intervention measures, there may be some degree of bias, particularly regarding placebo effects and patient expectations, which could interfere with subjective evaluations of treatment outcomes. Moreover, this study does not include a sham acupuncture control, which further increases the potential for bias, as the lack of a placebo control for acupuncture makes it difficult to rule out nonspecific treatment effects. Second, this study only included patients with flat-type ISSNHL, excluding the other 3 types of hearing curves, which may limit the generalizability of the results to a broader population of patients with ISSNHL with tinnitus.

### Conclusions

This pilot study evaluates the feasibility of combining EA with medication for flat-type ISSNHL, integrating both subjective and objective outcomes across multiple follow-up time points to assess potential benefits in hearing, tinnitus, anxiety, and quality of life. Using rigorous stratified randomization and high-quality data management, this study generates preliminary evidence on EA’s viability as a complementary therapy, laying the groundwork for the design and sample size estimation of future large-scale multicenter RCTs to advance nonpharmacological treatment options for ISSNHL.

## Appendix

Multimedia Appendix 1 CONSORT-eHEALTH checklist (V 1.6.1).

Multimedia Appendix 2 Case report form.

## Abbreviations

CACMS: China Academy of Chinese Medical Sciences
CONSORT: Consolidated Standards of Reporting Trials
CRF: case report form
EA: electroacupuncture
ENT: ear, nose, and throat
ISSNHL: idiopathic sudden sensorineural hearing loss
PTA: pure tone audiometry
RCT: randomized controlled trial
SAS: Self-Rating Anxiety Scale
SF-36: 36-Item Short Form Survey Questionnaire
SRT: speech-recognition thresholds in noise

## Data Availability

No data sets were generated or analyzed during this study. All relevant data from this study will be made available upon study completion and can be obtained by contacting the corresponding author.
