# Supplementary material for: Combination of Electroacupuncture and Medication for Patients With Tinnitus Associated With Idiopathic Sudden Sensorineural Hearing Loss: Protocol for a Prospective Randomized Controlled Trial
Source: JMIR Res Protoc. 2025 May 14;14:e69163. doi: 10.2196/69163 (PMC12120368; doi:10.2196/69163)
Supplement: Multimedia Appendix 2 [file resprot_v14i1e69163_app2.pdf]

ID Number:

A Randomized Controlled Pilot Clinical Trial on the Combination of Electroacupuncture and Medication for Patients With Tinnitus Associated With Idiopathic Sudden Sensorineural Hearing Loss

Case Report Form (CRF)

Subject Initials: |__|__|__|__|

Investigator Name:__________

Project Undertaken by: Institute of Acupuncture and Moxibustion, China Academy of Chinese Medical Sciences & Beijing Tongren Hospital, Capital Medical University

Instructions for Filling Out the Case Report Form (CRF)

1. Please fill in the relevant information clearly and legibly using a black gel pen or blue-black fountain pen in the appropriate fields.
2. The header on every page of the CRF must be completed in full.
3. When a question requires selecting an appropriate option, mark “×” in the corresponding box.

Example: ☒1 Yes, ☐ No.

4. All responses must be accurate and clear. The use of symbols such as “same as above” is not permitted.
5. Any modification to the records must not obscure the original entry. Draw a single horizontal line through the original data (e.g., ~~090mmHg~~), write the corrected value above it, and sign with the investigator’s initials (in uppercase) and the date.

Example:

- Incorrect entry: 2011/07/13 [year/month/day]
- Corrected format: 2011/07/~~13~~ 14 SJF 2011/07/14

The use of correction fluid or other methods to obscure the original data is strictly prohibited.

6. All required information must be filled in the designated spaces. If data is not collected or not applicable, use the following codes:
   - “ND” for Not Done
   - “NA” for Not Applicable
   - “UK” for Unknown

Add a brief explanation if needed (e.g., sample hemolyzed). If an entire page or section is not completed or not applicable, draw a line through it and mark as “ND” or “NA”.

7. Answer fields are formatted with a fixed number of spaces. If the answer has fewer digits, fill the leading spaces with zeroes.

Example: 56 kg should be entered as: **|0|5|6|.|0|0|**kg

Date：|__|__|__|__|__|__|__|__|

For hematology, serum biochemistry, and urinalysis results:

- Start entering data from the leftmost space
- Decimal points must be written in the provided space

8. The four-letter pinyin abbreviation of the subject's name must be fully filled:

    For two-character names, use the first two letters of each character's pinyin

    For three-character names, use the first letter of each character and the second letter of the third character's pinyin

    For four-character names, use the first letter of each character's pinyin

Examples:

- Zhang Fang → |Z|H|F|A|
- Li Zhengfen → |L|Z|F|E|
- Shangguan Xiaoyun → |S|G|X|Y|

9. Unless otherwise specified, all dates must be recorded accurately (year, month, and day), following the ISO 8601 format: yyyymmdd.

Example: December 21, 2007 → |2|0|0|7|year|1|2|month|2|1|day

If the exact date is unknown, provide known parts and use "UK" to fill the unknown day/month (do not leave blank).

Examples: 201010UK, 2010UKUK, UKUKUK

Time should be in 24-hour format. For example, use 21:00 instead of 09:00pm; record midnight as 00:00 (not 24:00) as the start of a new day.

10. Only the subject ID and pinyin initials should be recorded in the CRF. If outpatient/inpatient reports are to be pasted into the CRF, the subject's name must be fully covered in black ink and replaced with the subject ID and pinyin initials.
11. The investigator must carefully check each page of the CRF, sign, and date it.
12. All options are single-choice unless explicitly marked as "multiple choice".
13. During the clinical study, the Complications and Adverse Events/Reaction Forms

must be filled truthfully. In the event of a serious adverse event, the Serious Adverse Event Form must be completed and reported to the designated contact person at the project institution within 24 hours. If reported by phone, the form must be submitted afterward.

Institution (Center Name)   Contact Person   Phone Number

Institute of Acupuncture, China Academy of Chinese Medical Sciences,Huo Jin,13681158605

Beijing Tongren Hospital, Capital Medical University,Hao Xinping,13671218615

14. Investigator: the observing physician.

Overall procedure of this study

	Enrollment	Allocation	On-treatment follow-up		Post-treatment follow-up		
**Timepoint**	**$-t_1$**	**t_0**	**t_1**	**t_2**	**t_3**	**t_4**	**t_5**
**Enrollment**							
Eligibility screening	√						
Informed consent	√						
Allocation		√					
**Interventions**							
Control group			√	√			
EA group			√	√			
**Assessments**							
**Feasibility Outcomes**							
recruitment rate					√		
adherence rate					√		
data completeness					√		
Adverse Events			√	√	√	√	√
**Exploratory Outcomes**							
PTA[a]		√	√	√	√	√	√
Tinnitus Matching		√	√	√	√	√	√
THI[b]		√	√	√	√	√	√
**Secondary outcomes**							
SRT[c]		√	√	√	√	√	√
SAS[d]		√	√	√	√	√	√
SF-36[e]		√	√	√	√	√	√

[a]PTA: Pure tone audiometry

[b]THI: Tinnitus handicap inventory

[c]SRT: Speech-recognition thresholds in noise

Date：|__|__|__|__|__|__|__|__|

[d]SAS: Self-Rating Anxiety Scale

[e]SF-36: Short Form 36 Health Survey Questionnaire

Date：|__|__|__|__|__|__|__|__|

Date of Signing the Informed Consent Form: |__|__|__|__|__|__|__|__| (YYYY/MM/DD)

Inclusion Criteria (If any item is “No,” the subject cannot be included in this study.):

Inclusion Criteria	Yes	No
1. Gender unrestricted, aged between 18–65 years	☐	☐
2. Meets the 2015 diagnostic criteria for sudden deafness with tinnitus as defined by the Chinese Medical Association	☐	☐
3. Pure tone hearing threshold ≥ 40 dB	☐	☐
4. Duration of disease within 3–14 days	☐	☐
5. Clear consciousness, normal cognition, normal communication	☐	☐
6. Subject or guardian agrees to sign the informed consent form	☐	☐

Exclusion Criteria (If any item is “Yes,” the subject cannot be included in this study.):

Exclusion Criteria	Yes	No
1. Tympanic membrane perforation, cerumen impaction, or confirmed middle ear disease	☐	☐
2. Other types of hearing loss, e.g., drug-induced or noise-induced	☐	☐
3. History of ear trauma, ear surgery, or outer/middle/inner ear malformation	☐	☐
4. Received medication or acupuncture in past 6 months, or allergy to treatment drugs	☐	☐
5. Pregnant or	☐	☐

breastfeeding		
6. Severe systemic or psychiatric illness	☐	☐
7. Participation in another trial	☐	☐

Demographic Characteristics

1. Date of Birth	|__|__|__|__|__|__|__|__| (YYYY/MM/DD)
2. Gender	☐ Male ☐ Female
3. Ethnicity	☐ Han ☐ Other: ______
4. Marital Status	☐ Unmarried ☐ Married ☐ Separated ☐ Divorced ☐ Widowed
5. Education Level	☐ No schooling ☐ Primary ☐ Junior high ☐ High school/Technical school ☐ College/Undergraduate ☐ Graduate and above
6. Occupation	___________

Disease Information

Symptom	Yes	No
1. Sudden hearing loss with tinnitus in the left ear	☐	☐
2. Sudden hearing loss with tinnitus in the right ear	☐	☐
3. Ear fullness or blockage	☐	☐

Visit 1: Baseline Observation Form for Patients With Tinnitus Associated With Idiopathic Sudden Sensorineural Hearing Loss

Date：|__|__|__|__|__|__|__|__|

Hearing Change Observation Indicators

1. Pure Tone Audiometry (PTA)

Right Ear

**(dB HL)**	**250 Hz**	**500 Hz**	**1k Hz**	**2k Hz**	**4k Hz**	**8k Hz**
Bone Conduction						
Air Conduction						

Left Ear

**(dB HL)**	**250 Hz**	**500 Hz**	**1k Hz**	**2k Hz**	**4k Hz**	**8k Hz**
Bone Conduction						
Air Conduction						

- **Air-Bone Gap**: ________.
- **Type of Hearing Loss**:
  - High-frequency sloping
  - Low-frequency sloping
  - Flat
  - Profound
- **Degree of Hearing Loss**:
  - Moderate
  - Moderately severe
  - Severe
  - Profound

2. Speech Recognition Threshold Test in Noise (SRT)

Ear	Speech Recognition Threshold	Test Intensity (dB HL)	Test Material	Speech Recognition Score (%)
Right Ear			Sentences	
Left Ear			Sentences	

Attached Audiogram:

Tinnitus Change Observation Indicators

1. Tinnitus Matching

Please identify the sound frequency and loudness closest to your tinnitus.

Parameter	Options
**Tinnitus Frequency**	[ ] Low-frequency [ ] Mid-frequency [ ] High-frequency
**Tinnitus Loudness**	________

2. Tinnitus Handicap Inventory (THI)

No.	Question	0 Points (Yes)	2 Points (Sometimes)	4 Points (No)
1	Does tinnitus make it difficult for you to concentrate?	[ ]	[ ]	[ ]
2	Does the loudness of your tinnitus make it hard to hear others?	[ ]	[ ]	[ ]
3	Does tinnitus make you angry?	[ ]	[ ]	[ ]

No.	Question	0 Points (Yes)	2 Points (Sometimes)	4 Points (No)
4	Does tinnitus make you feel confused (irritated)?	[ ]	[ ]	[ ]
5	Does tinnitus make you feel desperate?	[ ]	[ ]	[ ]
6	Do you often complain about your tinnitus?	[ ]	[ ]	[ ]
7	Does tinnitus make it difficult to fall asleep at night?	[ ]	[ ]	[ ]
8	Do you feel unable to escape your tinnitus?	[ ]	[ ]	[ ]
9	Does tinnitus interfere with your social activities (e.g., dining out, watching movies, playing cards, or gatherings with friends)?	[ ]	[ ]	[ ]
10	Does tinnitus make you feel frustrated?	[ ]	[ ]	[ ]
11	Do you think tinnitus is a terrible condition?	[ ]	[ ]	[ ]
12	Does tinnitus make it hard to enjoy life?	[ ]	[ ]	[ ]
13	Does tinnitus interfere with your work or household chores?	[ ]	[ ]	[ ]
14	Does tinnitus make you easily irritable?	[ ]	[ ]	[ ]
15	Does tinnitus make reading or focusing on tasks difficult?	[ ]	[ ]	[ ]
16	Does tinnitus make you upset?	[ ]	[ ]	[ ]
17	Does tinnitus strain your relationships with friends or family?	[ ]	[ ]	[ ]
18	Is it difficult to shift your attention from tinnitus to other things?	[ ]	[ ]	[ ]
19	Do you feel unable to control your tinnitus?	[ ]	[ ]	[ ]

Date：|__|__|__|__|__|__|__|__|

No.	Question	0 Points (Yes)	2 Points (Sometimes)	4 Points (No)
20	Does tinnitus often make you feel fatigued?	[ ]	[ ]	[ ]
21	Does tinnitus make you feel depressed (lack interest in activities)?	[ ]	[ ]	[ ]
22	Does tinnitus make you feel anxious?	[ ]	[ ]	[ ]
23	Do you feel helpless about your tinnitus?	[ ]	[ ]	[ ]
24	Does tinnitus worsen under stress (e.g., during exams)?	[ ]	[ ]	[ ]
25	Does tinnitus make you feel insecure (unstable or unsafe)?	[ ]	[ ]	[ ]

Total Score: _________.

THI Grading:

Score Range	Grade	Description
1–16	Grade I	Mild tinnitus
18–36	Grade II	Slight tinnitus
38–56	Grade III	Moderate tinnitus
58–76	Grade IV	Severe tinnitus
78–100	Grade V	Catastrophic tinnitus

Self-Rating Anxiety Scale (SAS)

No.	Assessment Content	None or Rarely (1)	Sometimes (2)	Most of the Time (3)	Almost Always or Always (4)	Score
1	I feel more nervous and anxious than usual (Anxiety)	[ ] 1	[ ] 2	[ ] 3	[ ] 4	

Date：|__|__|__|__|__|__|__|__|

No.	Assessment Content	None or Rarely (1)	Sometimes (2)	Most of the Time (3)	Almost Always or Always (4)	Score
2	I feel afraid for no reason (Fear)	1	2	3	4	
3	I easily feel upset or panicked (Panic)	1	2	3	4	
4	I feel like I might go crazy (Sense of going crazy)	1	2	3	4	
*5	I feel that everything is fine and nothing bad will happen (Sense of impending misfortune)	1	2	3	4	
6	My hands and feet tremble or shake (Trembling)	1	2	3	4	
7	I am troubled by headaches, neck pain, or back pain (Physical pain)	1	2	3	4	
8	I feel weak and easily fatigued (Fatigue)	1	2	3	4	
*9	I feel calm and can sit still easily (Inability to sit still)	1	2	3	4	
10	I feel my heart beating fast (Palpitations)	1	2	3	4	
11	I am troubled by bouts of dizziness (Dizziness)	1	2	3	4	
12	I have fainting spells or feel like I might faint (Fainting sensation)	1	2	3	4	

Date：|__|__|__|__|__|__|__|__|

No.	Assessment Content	None or Rarely (1)	Sometimes (2)	Most of the Time (3)	Almost Always or Always (4)	Score
13	I can breathe in and out easily (Difficulty breathing)	1	2	3	4	
14	I feel numbness or tingling in my hands or feet (Numbness or tingling)	1	2	3	4	
15	I am troubled by stomach pain or indigestion (Stomach pain or indigestion)	1	2	3	4	
16	I often need to urinate (Frequent urination)	1	2	3	4	
17	My hands are usually dry and warm (Excessive sweating)	1	2	3	4	
18	My face feels flushed or hot (Facial flushing)	1	2	3	4	
19	I fall asleep easily and sleep well through the night (Sleep disturbance)	1	2	3	4	
20	I have nightmares	1	2	3	4	

Total Score: ________.

Note:

- Scoring uses a 1–4 scale. Items marked with * (5, 9, 13, 17, 19) are reverse-scored (4–1).
- Sum the scores of all 20 items to get the total score. Multiply the total score by 1.25, then round to the nearest integer to obtain the **standard score**.
- **Anxiety Evaluation Cutoff**:
    - Standard score ⩾ 50 indicates anxiety.

Date：|__|__|__|__|__|__|__|__|

    - 50–59: Mild anxiety
    - 60–69: Moderate anxiety
    - ⩾ 70: Severe anxiety
- Higher scores indicate a stronger tendency toward anxiety.

SF-36 Health Survey (Quality of Life Assessment)

1. General Health

**Question**	**Options (Circle the most appropriate situation)**
1. In general, your health is:	① Excellent ② Very good ③ Good ④ Fair ⑤ Poor
2. Compared to one year ago, how would you rate your health?	① Much better than one year ago ② Somewhat better than one year ago ③ About the same as one year ago ④ Somewhat worse than one year ago ⑤ Much worse than one year ago

3. Physical Functioning

The following questions relate to daily activities. Consider whether your health limits these activities and to what extent.

**Activity**	**1 (Greatly limited)**	**2 (Somewhat limited)**	**3 (Not limited)**
(1) Vigorous activities (e.g., running, lifting heavy objects, strenuous sports)	①	②	③
(2) Moderate activities (e.g., moving a table, sweeping, Tai Chi, simple exercises)	①	②	③
(3) Carrying groceries (e.g., shopping for vegetables)	①	②	③
(4) Climbing several flights of stairs	①	②	③
(5) Climbing one flight of stairs	①	②	③

Activity	1 (Greatly limited)	2 (Somewhat limited)	3 (Not limited)
(6) Bending, kneeling, or squatting	①	②	③
(7) Walking more than 1500 meters	①	②	③
(8) Walking 1000 meters	①	②	③
(9) Walking 100 meters	①	②	③
(10) Bathing or dressing yourself	①	②	③

4. Role Limitations Due to Physical Health

In the past 4 weeks, have you experienced the following problems with work or daily activities due to physical health?

Problem	1 (Yes)	2 (No)
(1) Cut down on the amount of time spent on work or other activities	①	②
(2) Accomplished less than you would like	①	②
(3) Were limited in the kind of work or activities	①	②
(4) Had difficulty performing work or other activities (e.g., required extra effort)	①	②

5. Role Limitations Due to Emotional Problems

In the past 4 weeks, have you experienced the following problems with work or daily activities due to emotional problems (e.g., feeling depressed or anxious)?

Problem	1 (Yes)	2 (No)
(1) Cut down on the amount of time spent on work or other activities	①	②
(2) Accomplished less than you would like	①	②
(3) Did work or activities less carefully than usual	①	②

6. Social Functioning

Question	Options
In the past 4 weeks, to what extent has your physical health or emotional problems interfered with your normal social activities with family, friends, neighbors, or groups?	① Not at all ② Slightly ③ Moderately ④ Quite a bit ⑤ Extremely

7. Bodily Pain

Question	Options
In the past 4 weeks, have you had bodily pain?	① None ② Very mild ③ Moderate ④ Severe ⑤ Very severe

8. Pain Interference

Question	Options
In the past 4 weeks, how much did pain interfere with your work or household chores?	① Not at all ② Slightly ③ Moderately ④ Quite a bit ⑤ Extremely

9. Mental Health

The following questions are about how you felt during the past 4 weeks. For each item, indicate how often you experienced it.

Feeling	6 (All of the time)	5 (Most of the time)	4 (A good bit of the time)	3 (Some of the time)	2 (A little of the time)	1 (None of the time)
(1) Felt full of life	①	②	③	④	⑤	⑥
(2) Been a very nervous person	①	②	③	④	⑤	⑥
(3) Felt so down that nothing could cheer you up	①	②	③	④	⑤	⑥
(4) Felt calm and peaceful	①	②	③	④	⑤	⑥
(5) Had a lot of energy	①	②	③	④	⑤	⑥

Feeling	6 (All of the time)	5 (Most of the time)	4 (A good bit of the time)	3 (Some of the time)	2 (A little of the time)	1 (None of the time)
(6) Felt downhearted and blue	①	②	③	④	⑤	⑥
(7) Felt worn out	①	②	③	④	⑤	⑥
(8) Been a happy person	①	②	③	④	⑤	⑥
(9) Felt tired	①	②	③	④	⑤	⑥

10. Social Functioning (Additional)

Question	Options
Poor health interfered with social activities (e.g., visiting relatives or friends)	① All of the time ② Most of the time ③ A good bit of the time ④ Some of the time ⑤ A little of the time ⑥ None of the time

11. General Health Perceptions

For each statement, indicate which answer best describes your situation.

Statement	1 (Definitely true)	2 (Mostly true)	3 (Not sure)	4 (Mostly false)	5 (Definitely false)
(1) I seem to get sick more easily than others	①	②	③	④	⑤
(2) I am as healthy as people around me	①	②	③	④	⑤
(3) I expect my health to get worse	①	②	③	④	⑤
(4) My health is excellent	①	②	③	④	⑤

Total Score: _________.

Scoring Formula:

Final score = [(Actual score - Lowest possible score) / (Highest possible score - Lowest

possible score)] × 100.
Each domain is scored out of 100, with higher scores indicating better quality of life in that domain.

Ear Fullness Sensation Assessment

Has your sensation of ear fullness improved? Check (√) the appropriate option:

**Option**	**Check (√)**
No improvement in ear fullness	[ ]
Ear fullness improved but worsens with activity or fatigue	[ ]
Ear fullness significantly improved	[ ]
Ear fullness completely resolved	[ ]

Visit 2

(Week 2 post-treatment)

Date：|__|__|__|__|__|__|__|__|

Hearing Change Observation Indicators

1. Pure Tone Audiometry (PTA)

Right Ear

(dB HL) 250 Hz 500 Hz 1k Hz 2k Hz 4k Hz 8k Hz

Bone Conduction

Air Conduction

Left Ear

(dB HL) 250 Hz 500 Hz 1k Hz 2k Hz 4k Hz 8k Hz

Bone Conduction

Air Conduction

- **Air-Bone Gap**: ________.
- **Type of Hearing Loss**:
  - High-frequency sloping
  - Low-frequency sloping
  - Flat
  - Profound
- **Degree of Hearing Loss**:
  - Moderate
  - Moderately severe
  - Severe
  - Profound

2. Speech Recognition Threshold Test in Noise (SRT)

Date：|__|__|__|__|__|__|__|__|

Ear	Speech Recognition Threshold	Test Intensity (dB HL)	Test Material	Speech Recognition Score (%)
Right Ear			Sentences	
Left Ear			Sentences	

Attached Audiogram:

Tinnitus Change Observation Indicators

1. Tinnitus Matching

Please identify the sound frequency and loudness closest to your tinnitus.

Parameter	Options
**Tinnitus Frequency**	[ ] Low-frequency [ ] Mid-frequency [ ] High-frequency
**Tinnitus Loudness**	_________

2. Tinnitus Handicap Inventory (THI)

No.	Question	0 Points (Yes)	2 Points (Sometimes)	4 Points (No)
1	Does tinnitus make it difficult for you to concentrate?	[ ]	[ ]	[ ]
2	Does the loudness of your tinnitus make it hard to hear others?	[ ]	[ ]	[ ]
3	Does tinnitus make you angry?	[ ]	[ ]	[ ]

Date：|__|__|__|__|__|__|__|__|

No.	Question	0 Points (Yes)	2 Points (Sometimes)	4 Points (No)
4	Does tinnitus make you feel confused (irritated)?	[ ]	[ ]	[ ]
5	Does tinnitus make you feel desperate?	[ ]	[ ]	[ ]
6	Do you often complain about your tinnitus?	[ ]	[ ]	[ ]
7	Does tinnitus make it difficult to fall asleep at night?	[ ]	[ ]	[ ]
8	Do you feel unable to escape your tinnitus?	[ ]	[ ]	[ ]
9	Does tinnitus interfere with your social activities (e.g., dining out, watching movies, playing cards, or gatherings with friends)?	[ ]	[ ]	[ ]
10	Does tinnitus make you feel frustrated?	[ ]	[ ]	[ ]
11	Do you think tinnitus is a terrible condition?	[ ]	[ ]	[ ]
12	Does tinnitus make it hard to enjoy life?	[ ]	[ ]	[ ]
13	Does tinnitus interfere with your work or household chores?	[ ]	[ ]	[ ]
14	Does tinnitus make you easily irritable?	[ ]	[ ]	[ ]
15	Does tinnitus make reading or focusing on tasks difficult?	[ ]	[ ]	[ ]
16	Does tinnitus make you upset?	[ ]	[ ]	[ ]
17	Does tinnitus strain your relationships with friends or family?	[ ]	[ ]	[ ]
18	Is it difficult to shift your attention from tinnitus to other things?	[ ]	[ ]	[ ]
19	Do you feel unable to control your tinnitus?	[ ]	[ ]	[ ]

Date：|__|__|__|__|__|__|__|__|

No.	Question	0 Points (Yes)	2 Points (Sometimes)	4 Points (No)
20	Does tinnitus often make you feel fatigued?	[ ]	[ ]	[ ]
21	Does tinnitus make you feel depressed (lack interest in activities)?	[ ]	[ ]	[ ]
22	Does tinnitus make you feel anxious?	[ ]	[ ]	[ ]
23	Do you feel helpless about your tinnitus?	[ ]	[ ]	[ ]
24	Does tinnitus worsen under stress (e.g., during exams)?	[ ]	[ ]	[ ]
25	Does tinnitus make you feel insecure (unstable or unsafe)?	[ ]	[ ]	[ ]

Total Score: _________.

THI Grading:

Score Range	Grade	Description
1–16	Grade I	Mild tinnitus
18–36	Grade II	Slight tinnitus
38–56	Grade III	Moderate tinnitus
58–76	Grade IV	Severe tinnitus
78–100	Grade V	Catastrophic tinnitus

Self-Rating Anxiety Scale (SAS)

No.	Assessment Content	None or Rarely (1)	Sometimes (2)	Most of the Time (3)	Almost Always or Always (4)	Score
1	I feel more nervous and anxious than usual (Anxiety)	[ ] 1	[ ] 2	[ ] 3	[ ] 4	

No.	Assessment Content	None or Rarely (1)	Sometimes (2)	Most of the Time (3)	Almost Always or Always (4)	Score
2	I feel afraid for no reason (Fear)	1	2	3	4	
3	I easily feel upset or panicked (Panic)	1	2	3	4	
4	I feel like I might go crazy (Sense of going crazy)	1	2	3	4	
*5	I feel that everything is fine and nothing bad will happen (Sense of impending misfortune)	1	2	3	4	
6	My hands and feet tremble or shake (Trembling)	1	2	3	4	
7	I am troubled by headaches, neck pain, or back pain (Physical pain)	1	2	3	4	
8	I feel weak and easily fatigued (Fatigue)	1	2	3	4	
*9	I feel calm and can sit still easily (Inability to sit still)	1	2	3	4	
10	I feel my heart beating fast (Palpitations)	1	2	3	4	
11	I am troubled by bouts of dizziness (Dizziness)	1	2	3	4	
12	I have fainting spells or feel like I might faint (Fainting sensation)	1	2	3	4	

Date：|__|__|__|__|__|__|__|__|

No.	Assessment Content	None or Rarely (1)	Sometimes (2)	Most of the Time (3)	Almost Always or Always (4)	Score
13	I can breathe in and out easily (Difficulty breathing)	1	2	3	4	
14	I feel numbness or tingling in my hands or feet (Numbness or tingling)	1	2	3	4	
15	I am troubled by stomach pain or indigestion (Stomach pain or indigestion)	1	2	3	4	
16	I often need to urinate (Frequent urination)	1	2	3	4	
17	My hands are usually dry and warm (Excessive sweating)	1	2	3	4	
18	My face feels flushed or hot (Facial flushing)	1	2	3	4	
19	I fall asleep easily and sleep well through the night (Sleep disturbance)	1	2	3	4	
20	I have nightmares	1	2	3	4	

Total Score: ________.

Note:

- Scoring uses a 1–4 scale. Items marked with * (5, 9, 13, 17, 19) are reverse-scored (4–1).
- Sum the scores of all 20 items to get the total score. Multiply the total score by 1.25, then round to the nearest integer to obtain the **standard score**.
- **Anxiety Evaluation Cutoff**:
  - Standard score ⩾ 50 indicates anxiety.

Date：|__|__|__|__|__|__|__|__|

- 50–59: Mild anxiety
- 60–69: Moderate anxiety
- ⩾ 70: Severe anxiety

- Higher scores indicate a stronger tendency toward anxiety.

SF-36 Health Survey (Quality of Life Assessment)

1. General Health

Question	Options (Circle the most appropriate situation)
1. In general, your health is:	① Excellent ② Very good ③ Good ④ Fair ⑤ Poor
2. Compared to one year ago, how would you rate your health?	① Much better than one year ago ② Somewhat better than one year ago ③ About the same as one year ago ④ Somewhat worse than one year ago ⑤ Much worse than one year ago

3. Physical Functioning

The following questions relate to daily activities. Consider whether your health limits these activities and to what extent.

Activity	1 (Greatly limited)	2 (Somewhat limited)	3 (Not limited)
(1) Vigorous activities (e.g., running, lifting heavy objects, strenuous sports)	①	②	③
(2) Moderate activities (e.g., moving a table, sweeping, Tai Chi, simple exercises)	①	②	③
(3) Carrying groceries (e.g., shopping for vegetables)	①	②	③
(4) Climbing several flights of stairs	①	②	③
(5) Climbing one flight of stairs	①	②	③

Date：|__|__|__|__|__|__|__|__|

Activity	1 (Greatly limited)	2 (Somewhat limited)	3 (Not limited)
(6) Bending, kneeling, or squatting	①	②	③
(7) Walking more than 1500 meters	①	②	③
(8) Walking 1000 meters	①	②	③
(9) Walking 100 meters	①	②	③
(10) Bathing or dressing yourself	①	②	③

4. Role Limitations Due to Physical Health

In the past 4 weeks, have you experienced the following problems with work or daily activities due to physical health?

Problem	1 (Yes)	2 (No)
(1) Cut down on the amount of time spent on work or other activities	①	②
(2) Accomplished less than you would like	①	②
(3) Were limited in the kind of work or activities	①	②
(4) Had difficulty performing work or other activities (e.g., required extra effort)	①	②

5. Role Limitations Due to Emotional Problems

In the past 4 weeks, have you experienced the following problems with work or daily activities due to emotional problems (e.g., feeling depressed or anxious)?

Problem	1 (Yes)	2 (No)
(1) Cut down on the amount of time spent on work or other activities	①	②
(2) Accomplished less than you would like	①	②
(3) Did work or activities less carefully than usual	①	②

6. Social Functioning

Question	Options
In the past 4 weeks, to what extent has your physical health or emotional problems interfered with your normal social activities with family, friends, neighbors, or groups?	① Not at all ② Slightly ③ Moderately ④ Quite a bit ⑤ Extremely

7. Bodily Pain

Question	Options
In the past 4 weeks, have you had bodily pain?	① None ② Very mild ③ Moderate ④ Severe ⑤ Very severe

8. Pain Interference

Question	Options
In the past 4 weeks, how much did pain interfere with your work or household chores?	① Not at all ② Slightly ③ Moderately ④ Quite a bit ⑤ Extremely

9. Mental Health

The following questions are about how you felt during the past 4 weeks. For each item, indicate how often you experienced it.

Feeling	6 (All of the time)	5 (Most of the time)	4 (A good bit of the time)	3 (Some of the time)	2 (A little of the time)	1 (None of the time)
(1) Felt full of life	①	②	③	④	⑤	⑥
(2) Been a very nervous person	①	②	③	④	⑤	⑥
(3) Felt so down that nothing could cheer you up	①	②	③	④	⑤	⑥
(4) Felt calm and peaceful	①	②	③	④	⑤	⑥
(5) Had a lot of energy	①	②	③	④	⑤	⑥

Date：|__|__|__|__|__|__|__|__|

Feeling	6 (All of the time)	5 (Most of the time)	4 (A good bit of the time)	3 (Some of the time)	2 (A little of the time)	1 (None of the time)
(6) Felt downhearted and blue	①	②	③	④	⑤	⑥
(7) Felt worn out	①	②	③	④	⑤	⑥
(8) Been a happy person	①	②	③	④	⑤	⑥
(9) Felt tired	①	②	③	④	⑤	⑥

10. Social Functioning (Additional)

Question	Options
Poor health interfered with social activities (e.g., visiting relatives or friends)	① All of the time ② Most of the time ③ A good bit of the time ④ Some of the time ⑤ A little of the time ⑥ None of the time

11. General Health Perceptions

For each statement, indicate which answer best describes your situation.

Statement	1 (Definitely true)	2 (Mostly true)	3 (Not sure)	4 (Mostly false)	5 (Definitely false)
(1) I seem to get sick more easily than others	①	②	③	④	⑤
(2) I am as healthy as people around me	①	②	③	④	⑤
(3) I expect my health to get worse	①	②	③	④	⑤
(4) My health is excellent	①	②	③	④	⑤

Total Score: _________.

Scoring Formula:

Final score = [(Actual score - Lowest possible score) / (Highest possible score - Lowest

possible score)] × 100.
Each domain is scored out of 100, with higher scores indicating better quality of life in that domain.

Ear Fullness Sensation Assessment

Has your sensation of ear fullness improved? Check (√) the appropriate option:

Option	Check (√)
No improvement in ear fullness	[ ]
Ear fullness improved but worsens with activity or fatigue	[ ]
Ear fullness significantly improved	[ ]
Ear fullness completely resolved	[ ]

Visit 3

(Week 4 post-treatment)

Date：|__|__|__|__|__|__|__|__|

Hearing Change Observation Indicators

1. Pure Tone Audiometry (PTA)

Right Ear

(dB HL)	250 Hz	500 Hz	1k Hz	2k Hz	4k Hz	8k Hz
Bone Conduction						
Air Conduction						

Left Ear

(dB HL)	250 Hz	500 Hz	1k Hz	2k Hz	4k Hz	8k Hz
Bone Conduction						
Air Conduction						

- **Air-Bone Gap**: _________.
- **Type of Hearing Loss**:
  - High-frequency sloping
  - Low-frequency sloping
  - Flat
  - Profound
- **Degree of Hearing Loss**:
  - Moderate
  - Moderately severe
  - Severe
  - Profound

2. Speech Recognition Threshold Test in Noise (SRT)

Ear	Speech Recognition Threshold	Test Intensity (dB HL)	Test Material	Speech Recognition Score (%)
Right Ear			Sentences	
Left Ear			Sentences	

Attached Audiogram:

Tinnitus Change Observation Indicators

1. Tinnitus Matching

Please identify the sound frequency and loudness closest to your tinnitus.

Parameter	Options
**Tinnitus Frequency**	[ ] Low-frequency [ ] Mid-frequency [ ] High-frequency
**Tinnitus Loudness**	________

2. Tinnitus Handicap Inventory (THI)

No.	Question	0 Points (Yes)	2 Points (Sometimes)	4 Points (No)
1	Does tinnitus make it difficult for you to concentrate?	[ ]	[ ]	[ ]
2	Does the loudness of your tinnitus make it hard to hear others?	[ ]	[ ]	[ ]
3	Does tinnitus make you angry?	[ ]	[ ]	[ ]

Date：|__|__|__|__|__|__|__|__|

No.	Question	0 Points (Yes)	2 Points (Sometimes)	4 Points (No)
4	Does tinnitus make you feel confused (irritated)?	[ ]	[ ]	[ ]
5	Does tinnitus make you feel desperate?	[ ]	[ ]	[ ]
6	Do you often complain about your tinnitus?	[ ]	[ ]	[ ]
7	Does tinnitus make it difficult to fall asleep at night?	[ ]	[ ]	[ ]
8	Do you feel unable to escape your tinnitus?	[ ]	[ ]	[ ]
9	Does tinnitus interfere with your social activities (e.g., dining out, watching movies, playing cards, or gatherings with friends)?	[ ]	[ ]	[ ]
10	Does tinnitus make you feel frustrated?	[ ]	[ ]	[ ]
11	Do you think tinnitus is a terrible condition?	[ ]	[ ]	[ ]
12	Does tinnitus make it hard to enjoy life?	[ ]	[ ]	[ ]
13	Does tinnitus interfere with your work or household chores?	[ ]	[ ]	[ ]
14	Does tinnitus make you easily irritable?	[ ]	[ ]	[ ]
15	Does tinnitus make reading or focusing on tasks difficult?	[ ]	[ ]	[ ]
16	Does tinnitus make you upset?	[ ]	[ ]	[ ]
17	Does tinnitus strain your relationships with friends or family?	[ ]	[ ]	[ ]
18	Is it difficult to shift your attention from tinnitus to other things?	[ ]	[ ]	[ ]
19	Do you feel unable to control your tinnitus?	[ ]	[ ]	[ ]

Date：|__|__|__|__|__|__|__|__|

No.	Question	0 Points (Yes)	2 Points (Sometimes)	4 Points (No)
20	Does tinnitus often make you feel fatigued?	[ ]	[ ]	[ ]
21	Does tinnitus make you feel depressed (lack interest in activities)?	[ ]	[ ]	[ ]
22	Does tinnitus make you feel anxious?	[ ]	[ ]	[ ]
23	Do you feel helpless about your tinnitus?	[ ]	[ ]	[ ]
24	Does tinnitus worsen under stress (e.g., during exams)?	[ ]	[ ]	[ ]
25	Does tinnitus make you feel insecure (unstable or unsafe)?	[ ]	[ ]	[ ]

Total Score: _________.

THI Grading:

Score Range	Grade	Description
1–16	Grade I	Mild tinnitus
18–36	Grade II	Slight tinnitus
38–56	Grade III	Moderate tinnitus
58–76	Grade IV	Severe tinnitus
78–100	Grade V	Catastrophic tinnitus

Self-Rating Anxiety Scale (SAS)

No.	Assessment Content	None or Rarely (1)	Sometimes (2)	Most of the Time (3)	Almost Always or Always (4)	Score
1	I feel more nervous and anxious than usual (Anxiety)	[ ] 1	[ ] 2	[ ] 3	[ ] 4	

No.	Assessment Content	None or Rarely (1)	Sometimes (2)	Most of the Time (3)	Almost Always or Always (4)	Score
2	I feel afraid for no reason (Fear)	1	2	3	4	
3	I easily feel upset or panicked (Panic)	1	2	3	4	
4	I feel like I might go crazy (Sense of going crazy)	1	2	3	4	
*5	I feel that everything is fine and nothing bad will happen (Sense of impending misfortune)	1	2	3	4	
6	My hands and feet tremble or shake (Trembling)	1	2	3	4	
7	I am troubled by headaches, neck pain, or back pain (Physical pain)	1	2	3	4	
8	I feel weak and easily fatigued (Fatigue)	1	2	3	4	
*9	I feel calm and can sit still easily (Inability to sit still)	1	2	3	4	
10	I feel my heart beating fast (Palpitations)	1	2	3	4	
11	I am troubled by bouts of dizziness (Dizziness)	1	2	3	4	
12	I have fainting spells or feel like I might faint (Fainting sensation)	1	2	3	4	

Date：|__|__|__|__|__|__|__|__|

No.	Assessment Content	None or Rarely (1)	Sometimes (2)	Most of the Time (3)	Almost Always or Always (4)	Score
13	I can breathe in and out easily (Difficulty breathing)	1	2	3	4	
14	I feel numbness or tingling in my hands or feet (Numbness or tingling)	1	2	3	4	
15	I am troubled by stomach pain or indigestion (Stomach pain or indigestion)	1	2	3	4	
16	I often need to urinate (Frequent urination)	1	2	3	4	
17	My hands are usually dry and warm (Excessive sweating)	1	2	3	4	
18	My face feels flushed or hot (Facial flushing)	1	2	3	4	
19	I fall asleep easily and sleep well through the night (Sleep disturbance)	1	2	3	4	
20	I have nightmares	1	2	3	4	

Total Score: ________.

Note:

- Scoring uses a 1–4 scale. Items marked with * (5, 9, 13, 17, 19) are reverse-scored (4–1).
- Sum the scores of all 20 items to get the total score. Multiply the total score by 1.25, then round to the nearest integer to obtain the **standard score**.
- **Anxiety Evaluation Cutoff**:
  - Standard score ⩾ 50 indicates anxiety.

Date：|__|__|__|__|__|__|__|__|

- 50–59: Mild anxiety
- 60–69: Moderate anxiety
- ⩾ 70: Severe anxiety

- Higher scores indicate a stronger tendency toward anxiety.

SF-36 Health Survey (Quality of Life Assessment)

1. General Health

Question	Options (Circle the most appropriate situation)
1. In general, your health is:	① Excellent ② Very good ③ Good ④ Fair ⑤ Poor
2. Compared to one year ago, how would you rate your health?	① Much better than one year ago ② Somewhat better than one year ago ③ About the same as one year ago ④ Somewhat worse than one year ago ⑤ Much worse than one year ago

3. Physical Functioning

The following questions relate to daily activities. Consider whether your health limits these activities and to what extent.

Activity	1 (Greatly limited)	2 (Somewhat limited)	3 (Not limited)
(1) Vigorous activities (e.g., running, lifting heavy objects, strenuous sports)	①	②	③
(2) Moderate activities (e.g., moving a table, sweeping, Tai Chi, simple exercises)	①	②	③
(3) Carrying groceries (e.g., shopping for vegetables)	①	②	③
(4) Climbing several flights of stairs	①	②	③
(5) Climbing one flight of stairs	①	②	③

Date：|__|__|__|__|__|__|__|__|

Activity	1 (Greatly limited)	2 (Somewhat limited)	3 (Not limited)
(6) Bending, kneeling, or squatting	①	②	③
(7) Walking more than 1500 meters	①	②	③
(8) Walking 1000 meters	①	②	③
(9) Walking 100 meters	①	②	③
(10) Bathing or dressing yourself	①	②	③

4. Role Limitations Due to Physical Health

In the past 4 weeks, have you experienced the following problems with work or daily activities due to physical health?

Problem	1 (Yes)	2 (No)
(1) Cut down on the amount of time spent on work or other activities	①	②
(2) Accomplished less than you would like	①	②
(3) Were limited in the kind of work or activities	①	②
(4) Had difficulty performing work or other activities (e.g., required extra effort)	①	②

5. Role Limitations Due to Emotional Problems

In the past 4 weeks, have you experienced the following problems with work or daily activities due to emotional problems (e.g., feeling depressed or anxious)?

Problem	1 (Yes)	2 (No)
(1) Cut down on the amount of time spent on work or other activities	①	②
(2) Accomplished less than you would like	①	②
(3) Did work or activities less carefully than usual	①	②

6. Social Functioning

Question	Options
In the past 4 weeks, to what extent has your physical health or emotional problems interfered with your normal social activities with family, friends, neighbors, or groups?	① Not at all ② Slightly ③ Moderately ④ Quite a bit ⑤ Extremely

7. Bodily Pain

Question	Options
In the past 4 weeks, have you had bodily pain?	① None ② Very mild ③ Moderate ④ Severe ⑤ Very severe

8. Pain Interference

Question	Options
In the past 4 weeks, how much did pain interfere with your work or household chores?	① Not at all ② Slightly ③ Moderately ④ Quite a bit ⑤ Extremely

9. Mental Health

The following questions are about how you felt during the past 4 weeks. For each item, indicate how often you experienced it.

Feeling	6 (All of the time)	5 (Most of the time)	4 (A good bit of the time)	3 (Some of the time)	2 (A little of the time)	1 (None of the time)
(1) Felt full of life	①	②	③	④	⑤	⑥
(2) Been a very nervous person	①	②	③	④	⑤	⑥
(3) Felt so down that nothing could cheer you up	①	②	③	④	⑤	⑥
(4) Felt calm and peaceful	①	②	③	④	⑤	⑥
(5) Had a lot of energy	①	②	③	④	⑤	⑥

Date：|__|__|__|__|__|__|__|__|

Feeling	6 (All of the time)	5 (Most of the time)	4 (A good bit of the time)	3 (Some of the time)	2 (A little of the time)	1 (None of the time)
(6) Felt downhearted and blue	①	②	③	④	⑤	⑥
(7) Felt worn out	①	②	③	④	⑤	⑥
(8) Been a happy person	①	②	③	④	⑤	⑥
(9) Felt tired	①	②	③	④	⑤	⑥

10. Social Functioning (Additional)

Question	Options
Poor health interfered with social activities (e.g., visiting relatives or friends)	① All of the time ② Most of the time ③ A good bit of the time ④ Some of the time ⑤ A little of the time ⑥ None of the time

11. General Health Perceptions

For each statement, indicate which answer best describes your situation.

Statement	1 (Definitely true)	2 (Mostly true)	3 (Not sure)	4 (Mostly false)	5 (Definitely false)
(1) I seem to get sick more easily than others	①	②	③	④	⑤
(2) I am as healthy as people around me	①	②	③	④	⑤
(3) I expect my health to get worse	①	②	③	④	⑤
(4) My health is excellent	①	②	③	④	⑤

Total Score: _________.

Scoring Formula:

Final score = [(Actual score - Lowest possible score) / (Highest possible score - Lowest

possible score)] × 100.
Each domain is scored out of 100, with higher scores indicating better quality of life in that domain.

Ear Fullness Sensation Assessment

Has your sensation of ear fullness improved? Check (√) the appropriate option:

Option	Check (√)
No improvement in ear fullness	[ ]
Ear fullness improved but worsens with activity or fatigue	[ ]
Ear fullness significantly improved	[ ]
Ear fullness completely resolved	[ ]

Visit 4

(Week 8 post-treatment)

Date：|__|__|__|__|__|__|__|__|

Hearing Change Observation Indicators

1. Pure Tone Audiometry (PTA)

Right Ear

**(dB HL)**	**250 Hz**	**500 Hz**	**1k Hz**	**2k Hz**	**4k Hz**	**8k Hz**
Bone Conduction						
Air Conduction						

Left Ear

**(dB HL)**	**250 Hz**	**500 Hz**	**1k Hz**	**2k Hz**	**4k Hz**	**8k Hz**
Bone Conduction						
Air Conduction						

- **Air-Bone Gap**: _________.
- **Type of Hearing Loss**:
  - High-frequency sloping
  - Low-frequency sloping
  - Flat
  - Profound
- **Degree of Hearing Loss**:
  - Moderate
  - Moderately severe
  - Severe
  - Profound

2. Speech Recognition Threshold Test in Noise (SRT)

Ear	Speech Recognition Threshold	Test Intensity (dB HL)	Test Material	Speech Recognition Score (%)
Right Ear			Sentences	
Left Ear			Sentences	

Attached Audiogram:

Tinnitus Change Observation Indicators

1. Tinnitus Matching

Please identify the sound frequency and loudness closest to your tinnitus.

Parameter	Options
**Tinnitus Frequency**	[ ] Low-frequency [ ] Mid-frequency [ ] High-frequency
**Tinnitus Loudness**	__________

2. Tinnitus Handicap Inventory (THI)

No.	Question	0 Points (Yes)	2 Points (Sometimes)	4 Points (No)
1	Does tinnitus make it difficult for you to concentrate?	[ ]	[ ]	[ ]
2	Does the loudness of your tinnitus make it hard to hear others?	[ ]	[ ]	[ ]
3	Does tinnitus make you angry?	[ ]	[ ]	[ ]

No.	Question	0 Points (Yes)	2 Points (Sometimes)	4 Points (No)
4	Does tinnitus make you feel confused (irritated)?	[ ]	[ ]	[ ]
5	Does tinnitus make you feel desperate?	[ ]	[ ]	[ ]
6	Do you often complain about your tinnitus?	[ ]	[ ]	[ ]
7	Does tinnitus make it difficult to fall asleep at night?	[ ]	[ ]	[ ]
8	Do you feel unable to escape your tinnitus?	[ ]	[ ]	[ ]
9	Does tinnitus interfere with your social activities (e.g., dining out, watching movies, playing cards, or gatherings with friends)?	[ ]	[ ]	[ ]
10	Does tinnitus make you feel frustrated?	[ ]	[ ]	[ ]
11	Do you think tinnitus is a terrible condition?	[ ]	[ ]	[ ]
12	Does tinnitus make it hard to enjoy life?	[ ]	[ ]	[ ]
13	Does tinnitus interfere with your work or household chores?	[ ]	[ ]	[ ]
14	Does tinnitus make you easily irritable?	[ ]	[ ]	[ ]
15	Does tinnitus make reading or focusing on tasks difficult?	[ ]	[ ]	[ ]
16	Does tinnitus make you upset?	[ ]	[ ]	[ ]
17	Does tinnitus strain your relationships with friends or family?	[ ]	[ ]	[ ]
18	Is it difficult to shift your attention from tinnitus to other things?	[ ]	[ ]	[ ]
19	Do you feel unable to control your tinnitus?	[ ]	[ ]	[ ]

Date：|__|__|__|__|__|__|__|__|

No.	Question	0 Points (Yes)	2 Points (Sometimes)	4 Points (No)
20	Does tinnitus often make you feel fatigued?	[ ]	[ ]	[ ]
21	Does tinnitus make you feel depressed (lack interest in activities)?	[ ]	[ ]	[ ]
22	Does tinnitus make you feel anxious?	[ ]	[ ]	[ ]
23	Do you feel helpless about your tinnitus?	[ ]	[ ]	[ ]
24	Does tinnitus worsen under stress (e.g., during exams)?	[ ]	[ ]	[ ]
25	Does tinnitus make you feel insecure (unstable or unsafe)?	[ ]	[ ]	[ ]

Total Score: _________.

THI Grading:

Score Range	Grade	Description
1–16	Grade I	Mild tinnitus
18–36	Grade II	Slight tinnitus
38–56	Grade III	Moderate tinnitus
58–76	Grade IV	Severe tinnitus
78–100	Grade V	Catastrophic tinnitus

Self-Rating Anxiety Scale (SAS)

No.	Assessment Content	None or Rarely (1)	Sometimes (2)	Most of the Time (3)	Almost Always or Always (4)	Score
1	I feel more nervous and anxious than usual (Anxiety)	[ ] 1	[ ] 2	[ ] 3	[ ] 4	

No.	Assessment Content	None or Rarely (1)	Sometimes (2)	Most of the Time (3)	Almost Always or Always (4)	Score
2	I feel afraid for no reason (Fear)	1	2	3	4	
3	I easily feel upset or panicked (Panic)	1	2	3	4	
4	I feel like I might go crazy (Sense of going crazy)	1	2	3	4	
*5	I feel that everything is fine and nothing bad will happen (Sense of impending misfortune)	1	2	3	4	
6	My hands and feet tremble or shake (Trembling)	1	2	3	4	
7	I am troubled by headaches, neck pain, or back pain (Physical pain)	1	2	3	4	
8	I feel weak and easily fatigued (Fatigue)	1	2	3	4	
*9	I feel calm and can sit still easily (Inability to sit still)	1	2	3	4	
10	I feel my heart beating fast (Palpitations)	1	2	3	4	
11	I am troubled by bouts of dizziness (Dizziness)	1	2	3	4	
12	I have fainting spells or feel like I might faint (Fainting sensation)	1	2	3	4	

Date：|__|__|__|__|__|__|__|__|

No.	Assessment Content	None or Rarely (1)	Sometimes (2)	Most of the Time (3)	Almost Always or Always (4)	Score
13	I can breathe in and out easily (Difficulty breathing)	1	2	3	4	
14	I feel numbness or tingling in my hands or feet (Numbness or tingling)	1	2	3	4	
15	I am troubled by stomach pain or indigestion (Stomach pain or indigestion)	1	2	3	4	
16	I often need to urinate (Frequent urination)	1	2	3	4	
17	My hands are usually dry and warm (Excessive sweating)	1	2	3	4	
18	My face feels flushed or hot (Facial flushing)	1	2	3	4	
19	I fall asleep easily and sleep well through the night (Sleep disturbance)	1	2	3	4	
20	I have nightmares	1	2	3	4	

Total Score: ________.

Note:

- Scoring uses a 1–4 scale. Items marked with * (5, 9, 13, 17, 19) are reverse-scored (4–1).
- Sum the scores of all 20 items to get the total score. Multiply the total score by 1.25, then round to the nearest integer to obtain the **standard score**.
- **Anxiety Evaluation Cutoff**:
  - Standard score ⩾ 50 indicates anxiety.

Date：|__|__|__|__|__|__|__|__|

- 50–59: Mild anxiety
- 60–69: Moderate anxiety
- ⩾ 70: Severe anxiety

- Higher scores indicate a stronger tendency toward anxiety.

SF-36 Health Survey (Quality of Life Assessment)

1. General Health

**Question**	**Options (Circle the most appropriate situation)**
1. In general, your health is:	① Excellent ② Very good ③ Good ④ Fair ⑤ Poor
2. Compared to one year ago, how would you rate your health?	① Much better than one year ago ② Somewhat better than one year ago ③ About the same as one year ago ④ Somewhat worse than one year ago ⑤ Much worse than one year ago

3. Physical Functioning

The following questions relate to daily activities. Consider whether your health limits these activities and to what extent.

**Activity**	**1 (Greatly limited)**	**2 (Somewhat limited)**	**3 (Not limited)**
(1) Vigorous activities (e.g., running, lifting heavy objects, strenuous sports)	①	②	③
(2) Moderate activities (e.g., moving a table, sweeping, Tai Chi, simple exercises)	①	②	③
(3) Carrying groceries (e.g., shopping for vegetables)	①	②	③
(4) Climbing several flights of stairs	①	②	③
(5) Climbing one flight of stairs	①	②	③

Activity	1 (Greatly limited)	2 (Somewhat limited)	3 (Not limited)
(6) Bending, kneeling, or squatting	①	②	③
(7) Walking more than 1500 meters	①	②	③
(8) Walking 1000 meters	①	②	③
(9) Walking 100 meters	①	②	③
(10) Bathing or dressing yourself	①	②	③

4. Role Limitations Due to Physical Health

In the past 4 weeks, have you experienced the following problems with work or daily activities due to physical health?

Problem	1 (Yes)	2 (No)
(1) Cut down on the amount of time spent on work or other activities	①	②
(2) Accomplished less than you would like	①	②
(3) Were limited in the kind of work or activities	①	②
(4) Had difficulty performing work or other activities (e.g., required extra effort)	①	②

5. Role Limitations Due to Emotional Problems

In the past 4 weeks, have you experienced the following problems with work or daily activities due to emotional problems (e.g., feeling depressed or anxious)?

Problem	1 (Yes)	2 (No)
(1) Cut down on the amount of time spent on work or other activities	①	②
(2) Accomplished less than you would like	①	②
(3) Did work or activities less carefully than usual	①	②

6. Social Functioning

Question	Options
In the past 4 weeks, to what extent has your physical health or emotional problems interfered with your normal social activities with family, friends, neighbors, or groups?	① Not at all ② Slightly ③ Moderately ④ Quite a bit ⑤ Extremely

7. Bodily Pain

Question	Options
In the past 4 weeks, have you had bodily pain?	① None ② Very mild ③ Moderate ④ Severe ⑤ Very severe

8. Pain Interference

Question	Options
In the past 4 weeks, how much did pain interfere with your work or household chores?	① Not at all ② Slightly ③ Moderately ④ Quite a bit ⑤ Extremely

9. Mental Health

The following questions are about how you felt during the past 4 weeks. For each item, indicate how often you experienced it.

Feeling	6 (All of the time)	5 (Most of the time)	4 (A good bit of the time)	3 (Some of the time)	2 (A little of the time)	1 (None of the time)
(1) Felt full of life	①	②	③	④	⑤	⑥
(2) Been a very nervous person	①	②	③	④	⑤	⑥
(3) Felt so down that nothing could cheer you up	①	②	③	④	⑤	⑥
(4) Felt calm and peaceful	①	②	③	④	⑤	⑥
(5) Had a lot of energy	①	②	③	④	⑤	⑥

Date：|__|__|__|__|__|__|__|__|

Feeling	6 (All of the time)	5 (Most of the time)	4 (A good bit of the time)	3 (Some of the time)	2 (A little of the time)	1 (None of the time)
(6) Felt downhearted and blue	①	②	③	④	⑤	⑥
(7) Felt worn out	①	②	③	④	⑤	⑥
(8) Been a happy person	①	②	③	④	⑤	⑥
(9) Felt tired	①	②	③	④	⑤	⑥

10. Social Functioning (Additional)

Question	Options
Poor health interfered with social activities (e.g., visiting relatives or friends)	① All of the time ② Most of the time ③ A good bit of the time ④ Some of the time ⑤ A little of the time ⑥ None of the time

11. General Health Perceptions

For each statement, indicate which answer best describes your situation.

Statement	1 (Definitely true)	2 (Mostly true)	3 (Not sure)	4 (Mostly false)	5 (Definitely false)
(1) I seem to get sick more easily than others	①	②	③	④	⑤
(2) I am as healthy as people around me	①	②	③	④	⑤
(3) I expect my health to get worse	①	②	③	④	⑤
(4) My health is excellent	①	②	③	④	⑤

Total Score: ________.

Scoring Formula:

Final score = [(Actual score - Lowest possible score) / (Highest possible score - Lowest

possible score)] × 100.
Each domain is scored out of 100, with higher scores indicating better quality of life in that domain.

Ear Fullness Sensation Assessment

Has your sensation of ear fullness improved? Check (√) the appropriate option:

**Option**	**Check (√)**
No improvement in ear fullness	[ ]
Ear fullness improved but worsens with activity or fatigue	[ ]
Ear fullness significantly improved	[ ]
Ear fullness completely resolved	[ ]

Date：|__|__|__|__|__|__|__|__|

Visit 5

(Week 12 post-treatment)

Date：|__|__|__|__|__|__|__|__|

Hearing Change Observation Indicators

1. Pure Tone Audiometry (PTA)

Right Ear

(dB HL)	250 Hz	500 Hz	1k Hz	2k Hz	4k Hz	8k Hz
Bone Conduction						
Air Conduction						

Left Ear

(dB HL)	250 Hz	500 Hz	1k Hz	2k Hz	4k Hz	8k Hz
Bone Conduction						
Air Conduction						

- **Air-Bone Gap**: ________.
- **Type of Hearing Loss**:
  - High-frequency sloping
  - Low-frequency sloping
  - Flat
  - Profound
- **Degree of Hearing Loss**:
  - Moderate
  - Moderately severe
  - Severe
  - Profound

2. Speech Recognition Threshold Test in Noise (SRT)

Ear	Speech Recognition Threshold	Test Intensity (dB HL)	Test Material	Speech Recognition Score (%)
Right Ear			Sentences	
Left Ear			Sentences	

Attached Audiogram:

Tinnitus Change Observation Indicators

1. Tinnitus Matching

Please identify the sound frequency and loudness closest to your tinnitus.

Parameter	Options
**Tinnitus Frequency**	[ ] Low-frequency [ ] Mid-frequency [ ] High-frequency
**Tinnitus Loudness**	________

2. Tinnitus Handicap Inventory (THI)

No.	Question	0 Points (Yes)	2 Points (Sometimes)	4 Points (No)
1	Does tinnitus make it difficult for you to concentrate?	[ ]	[ ]	[ ]
2	Does the loudness of your tinnitus make it hard to hear others?	[ ]	[ ]	[ ]
3	Does tinnitus make you angry?	[ ]	[ ]	[ ]

Date：|__|__|__|__|__|__|__|__|

No.	Question	0 Points (Yes)	2 Points (Sometimes)	4 Points (No)
4	Does tinnitus make you feel confused (irritated)?	[ ]	[ ]	[ ]
5	Does tinnitus make you feel desperate?	[ ]	[ ]	[ ]
6	Do you often complain about your tinnitus?	[ ]	[ ]	[ ]
7	Does tinnitus make it difficult to fall asleep at night?	[ ]	[ ]	[ ]
8	Do you feel unable to escape your tinnitus?	[ ]	[ ]	[ ]
9	Does tinnitus interfere with your social activities (e.g., dining out, watching movies, playing cards, or gatherings with friends)?	[ ]	[ ]	[ ]
10	Does tinnitus make you feel frustrated?	[ ]	[ ]	[ ]
11	Do you think tinnitus is a terrible condition?	[ ]	[ ]	[ ]
12	Does tinnitus make it hard to enjoy life?	[ ]	[ ]	[ ]
13	Does tinnitus interfere with your work or household chores?	[ ]	[ ]	[ ]
14	Does tinnitus make you easily irritable?	[ ]	[ ]	[ ]
15	Does tinnitus make reading or focusing on tasks difficult?	[ ]	[ ]	[ ]
16	Does tinnitus make you upset?	[ ]	[ ]	[ ]
17	Does tinnitus strain your relationships with friends or family?	[ ]	[ ]	[ ]
18	Is it difficult to shift your attention from tinnitus to other things?	[ ]	[ ]	[ ]
19	Do you feel unable to control your tinnitus?	[ ]	[ ]	[ ]

Date：|__|__|__|__|__|__|__|__|

No.	Question	0 Points (Yes)	2 Points (Sometimes)	4 Points (No)
20	Does tinnitus often make you feel fatigued?	[ ]	[ ]	[ ]
21	Does tinnitus make you feel depressed (lack interest in activities)?	[ ]	[ ]	[ ]
22	Does tinnitus make you feel anxious?	[ ]	[ ]	[ ]
23	Do you feel helpless about your tinnitus?	[ ]	[ ]	[ ]
24	Does tinnitus worsen under stress (e.g., during exams)?	[ ]	[ ]	[ ]
25	Does tinnitus make you feel insecure (unstable or unsafe)?	[ ]	[ ]	[ ]

Total Score: ________.

THI Grading:

Score Range	Grade	Description
1–16	Grade I	Mild tinnitus
18–36	Grade II	Slight tinnitus
38–56	Grade III	Moderate tinnitus
58–76	Grade IV	Severe tinnitus
78–100	Grade V	Catastrophic tinnitus

Self-Rating Anxiety Scale (SAS)

No.	Assessment Content	None or Rarely (1)	Sometimes (2)	Most of the Time (3)	Almost Always or Always (4)	Score
1	I feel more nervous and anxious than usual (Anxiety)	[ ] 1	[ ] 2	[ ] 3	[ ] 4	

Date：|__|__|__|__|__|__|__|__|

No.	Assessment Content	None or Rarely (1)	Sometimes (2)	Most of the Time (3)	Almost Always or Always (4)	Score
2	I feel afraid for no reason (Fear)	1	2	3	4	
3	I easily feel upset or panicked (Panic)	1	2	3	4	
4	I feel like I might go crazy (Sense of going crazy)	1	2	3	4	
*5	I feel that everything is fine and nothing bad will happen (Sense of impending misfortune)	1	2	3	4	
6	My hands and feet tremble or shake (Trembling)	1	2	3	4	
7	I am troubled by headaches, neck pain, or back pain (Physical pain)	1	2	3	4	
8	I feel weak and easily fatigued (Fatigue)	1	2	3	4	
*9	I feel calm and can sit still easily (Inability to sit still)	1	2	3	4	
10	I feel my heart beating fast (Palpitations)	1	2	3	4	
11	I am troubled by bouts of dizziness (Dizziness)	1	2	3	4	
12	I have fainting spells or feel like I might faint (Fainting sensation)	1	2	3	4	

Date：|__|__|__|__|__|__|__|__|

No.	Assessment Content	None or Rarely (1)	Sometimes (2)	Most of the Time (3)	Almost Always or Always (4)	Score
13	I can breathe in and out easily (Difficulty breathing)	1	2	3	4	
14	I feel numbness or tingling in my hands or feet (Numbness or tingling)	1	2	3	4	
15	I am troubled by stomach pain or indigestion (Stomach pain or indigestion)	1	2	3	4	
16	I often need to urinate (Frequent urination)	1	2	3	4	
17	My hands are usually dry and warm (Excessive sweating)	1	2	3	4	
18	My face feels flushed or hot (Facial flushing)	1	2	3	4	
19	I fall asleep easily and sleep well through the night (Sleep disturbance)	1	2	3	4	
20	I have nightmares	1	2	3	4	

Total Score: ________.

Note:

- Scoring uses a 1–4 scale. Items marked with * (5, 9, 13, 17, 19) are reverse-scored (4–1).
- Sum the scores of all 20 items to get the total score. Multiply the total score by 1.25, then round to the nearest integer to obtain the **standard score**.
- **Anxiety Evaluation Cutoff**:
    - Standard score ⩾ 50 indicates anxiety.

Date：|__|__|__|__|__|__|__|__|

- 50–59: Mild anxiety
- 60–69: Moderate anxiety
- ⩾ 70: Severe anxiety

- Higher scores indicate a stronger tendency toward anxiety.

SF-36 Health Survey (Quality of Life Assessment)

1. General Health

Question	Options (Circle the most appropriate situation)
1. In general, your health is:	① Excellent ② Very good ③ Good ④ Fair ⑤ Poor
2. Compared to one year ago, how would you rate your health?	① Much better than one year ago ② Somewhat better than one year ago ③ About the same as one year ago ④ Somewhat worse than one year ago ⑤ Much worse than one year ago

3. Physical Functioning

The following questions relate to daily activities. Consider whether your health limits these activities and to what extent.

Activity	1 (Greatly limited)	2 (Somewhat limited)	3 (Not limited)
(1) Vigorous activities (e.g., running, lifting heavy objects, strenuous sports)	①	②	③
(2) Moderate activities (e.g., moving a table, sweeping, Tai Chi, simple exercises)	①	②	③
(3) Carrying groceries (e.g., shopping for vegetables)	①	②	③
(4) Climbing several flights of stairs	①	②	③
(5) Climbing one flight of stairs	①	②	③

Activity	1 (Greatly limited)	2 (Somewhat limited)	3 (Not limited)
(6) Bending, kneeling, or squatting	①	②	③
(7) Walking more than 1500 meters	①	②	③
(8) Walking 1000 meters	①	②	③
(9) Walking 100 meters	①	②	③
(10) Bathing or dressing yourself	①	②	③

4. Role Limitations Due to Physical Health

In the past 4 weeks, have you experienced the following problems with work or daily activities due to physical health?

Problem	1 (Yes)	2 (No)
(1) Cut down on the amount of time spent on work or other activities	①	②
(2) Accomplished less than you would like	①	②
(3) Were limited in the kind of work or activities	①	②
(4) Had difficulty performing work or other activities (e.g., required extra effort)	①	②

5. Role Limitations Due to Emotional Problems

In the past 4 weeks, have you experienced the following problems with work or daily activities due to emotional problems (e.g., feeling depressed or anxious)?

Problem	1 (Yes)	2 (No)
(1) Cut down on the amount of time spent on work or other activities	①	②
(2) Accomplished less than you would like	①	②
(3) Did work or activities less carefully than usual	①	②

6. Social Functioning

Question	Options
In the past 4 weeks, to what extent has your physical health or emotional problems interfered with your normal social activities with family, friends, neighbors, or groups?	① Not at all ② Slightly ③ Moderately ④ Quite a bit ⑤ Extremely

7. Bodily Pain

Question	Options
In the past 4 weeks, have you had bodily pain?	① None ② Very mild ③ Moderate ④ Severe ⑤ Very severe

8. Pain Interference

Question	Options
In the past 4 weeks, how much did pain interfere with your work or household chores?	① Not at all ② Slightly ③ Moderately ④ Quite a bit ⑤ Extremely

9. Mental Health

The following questions are about how you felt during the past 4 weeks. For each item, indicate how often you experienced it.

Feeling	6 (All of the time)	5 (Most of the time)	4 (A good bit of the time)	3 (Some of the time)	2 (A little of the time)	1 (None of the time)
(1) Felt full of life	①	②	③	④	⑤	⑥
(2) Been a very nervous person	①	②	③	④	⑤	⑥
(3) Felt so down that nothing could cheer you up	①	②	③	④	⑤	⑥
(4) Felt calm and peaceful	①	②	③	④	⑤	⑥
(5) Had a lot of energy	①	②	③	④	⑤	⑥

Date：|__|__|__|__|__|__|__|__|

Feeling	6 (All of the time)	5 (Most of the time)	4 (A good bit of the time)	3 (Some of the time)	2 (A little of the time)	1 (None of the time)
(6) Felt downhearted and blue	①	②	③	④	⑤	⑥
(7) Felt worn out	①	②	③	④	⑤	⑥
(8) Been a happy person	①	②	③	④	⑤	⑥
(9) Felt tired	①	②	③	④	⑤	⑥

10. Social Functioning (Additional)

Question	Options
Poor health interfered with social activities (e.g., visiting relatives or friends)	① All of the time ② Most of the time ③ A good bit of the time ④ Some of the time ⑤ A little of the time ⑥ None of the time

11. General Health Perceptions

For each statement, indicate which answer best describes your situation.

Statement	1 (Definitely true)	2 (Mostly true)	3 (Not sure)	4 (Mostly false)	5 (Definitely false)
(1) I seem to get sick more easily than others	①	②	③	④	⑤
(2) I am as healthy as people around me	①	②	③	④	⑤
(3) I expect my health to get worse	①	②	③	④	⑤
(4) My health is excellent	①	②	③	④	⑤

Total Score: _________.

Scoring Formula:

Final score = [(Actual score - Lowest possible score) / (Highest possible score - Lowest

possible score)] × 100.
Each domain is scored out of 100, with higher scores indicating better quality of life in that domain.

Ear Fullness Sensation Assessment

Has your sensation of ear fullness improved? Check (√) the appropriate option:

Option	Check (√)
No improvement in ear fullness	[ ]
Ear fullness improved but worsens with activity or fatigue	[ ]
Ear fullness significantly improved	[ ]
Ear fullness completely resolved	[ ]

Visit 6

(Week 16 post-treatment)

Date：|__|__|__|__|__|__|__|__|

Hearing Change Observation Indicators

1. Pure Tone Audiometry (PTA)

Right Ear

(dB HL)	250 Hz	500 Hz	1k Hz	2k Hz	4k Hz	8k Hz
Bone Conduction						
Air Conduction						

Left Ear

(dB HL)	250 Hz	500 Hz	1k Hz	2k Hz	4k Hz	8k Hz
Bone Conduction						
Air Conduction						

- **Air-Bone Gap**: _________.
- **Type of Hearing Loss**:
  - High-frequency sloping
  - Low-frequency sloping
  - Flat
  - Profound
- **Degree of Hearing Loss**:
  - Moderate
  - Moderately severe
  - Severe
  - Profound

2. Speech Recognition Threshold Test in Noise (SRT)

Ear	Speech Recognition Threshold	Test Intensity (dB HL)	Test Material	Speech Recognition Score (%)
Right Ear			Sentences	
Left Ear			Sentences	

Attached Audiogram:

Tinnitus Change Observation Indicators

1. Tinnitus Matching

Please identify the sound frequency and loudness closest to your tinnitus.

Parameter	Options
**Tinnitus Frequency**	[ ] Low-frequency [ ] Mid-frequency [ ] High-frequency
**Tinnitus Loudness**	________

2. Tinnitus Handicap Inventory (THI)

No.	Question	0 Points (Yes)	2 Points (Sometimes)	4 Points (No)
1	Does tinnitus make it difficult for you to concentrate?	[ ]	[ ]	[ ]
2	Does the loudness of your tinnitus make it hard to hear others?	[ ]	[ ]	[ ]
3	Does tinnitus make you angry?	[ ]	[ ]	[ ]

Date：|__|__|__|__|__|__|__|__|

No.	Question	0 Points (Yes)	2 Points (Sometimes)	4 Points (No)
4	Does tinnitus make you feel confused (irritated)?	[ ]	[ ]	[ ]
5	Does tinnitus make you feel desperate?	[ ]	[ ]	[ ]
6	Do you often complain about your tinnitus?	[ ]	[ ]	[ ]
7	Does tinnitus make it difficult to fall asleep at night?	[ ]	[ ]	[ ]
8	Do you feel unable to escape your tinnitus?	[ ]	[ ]	[ ]
9	Does tinnitus interfere with your social activities (e.g., dining out, watching movies, playing cards, or gatherings with friends)?	[ ]	[ ]	[ ]
10	Does tinnitus make you feel frustrated?	[ ]	[ ]	[ ]
11	Do you think tinnitus is a terrible condition?	[ ]	[ ]	[ ]
12	Does tinnitus make it hard to enjoy life?	[ ]	[ ]	[ ]
13	Does tinnitus interfere with your work or household chores?	[ ]	[ ]	[ ]
14	Does tinnitus make you easily irritable?	[ ]	[ ]	[ ]
15	Does tinnitus make reading or focusing on tasks difficult?	[ ]	[ ]	[ ]
16	Does tinnitus make you upset?	[ ]	[ ]	[ ]
17	Does tinnitus strain your relationships with friends or family?	[ ]	[ ]	[ ]
18	Is it difficult to shift your attention from tinnitus to other things?	[ ]	[ ]	[ ]
19	Do you feel unable to control your tinnitus?	[ ]	[ ]	[ ]

Date：|__|__|__|__|__|__|__|__|

No.	Question	0 Points (Yes)	2 Points (Sometimes)	4 Points (No)
20	Does tinnitus often make you feel fatigued?	[ ]	[ ]	[ ]
21	Does tinnitus make you feel depressed (lack interest in activities)?	[ ]	[ ]	[ ]
22	Does tinnitus make you feel anxious?	[ ]	[ ]	[ ]
23	Do you feel helpless about your tinnitus?	[ ]	[ ]	[ ]
24	Does tinnitus worsen under stress (e.g., during exams)?	[ ]	[ ]	[ ]
25	Does tinnitus make you feel insecure (unstable or unsafe)?	[ ]	[ ]	[ ]

Total Score: _________.

THI Grading:

Score Range	Grade	Description
1–16	Grade I	Mild tinnitus
18–36	Grade II	Slight tinnitus
38–56	Grade III	Moderate tinnitus
58–76	Grade IV	Severe tinnitus
78–100	Grade V	Catastrophic tinnitus

Self-Rating Anxiety Scale (SAS)

No.	Assessment Content	None or Rarely (1)	Sometimes (2)	Most of the Time (3)	Almost Always or Always (4)	Score
1	I feel more nervous and anxious than usual (Anxiety)	[ ] 1	[ ] 2	[ ] 3	[ ] 4	

No.	Assessment Content	None or Rarely (1)	Sometimes (2)	Most of the Time (3)	Almost Always or Always (4)	Score
2	I feel afraid for no reason (Fear)	1	2	3	4	
3	I easily feel upset or panicked (Panic)	1	2	3	4	
4	I feel like I might go crazy (Sense of going crazy)	1	2	3	4	
*5	I feel that everything is fine and nothing bad will happen (Sense of impending misfortune)	1	2	3	4	
6	My hands and feet tremble or shake (Trembling)	1	2	3	4	
7	I am troubled by headaches, neck pain, or back pain (Physical pain)	1	2	3	4	
8	I feel weak and easily fatigued (Fatigue)	1	2	3	4	
*9	I feel calm and can sit still easily (Inability to sit still)	1	2	3	4	
10	I feel my heart beating fast (Palpitations)	1	2	3	4	
11	I am troubled by bouts of dizziness (Dizziness)	1	2	3	4	
12	I have fainting spells or feel like I might faint (Fainting sensation)	1	2	3	4	

Date：|__|__|__|__|__|__|__|__|

No.	Assessment Content	None or Rarely (1)	Sometimes (2)	Most of the Time (3)	Almost Always or Always (4)	Score
13	I can breathe in and out easily (Difficulty breathing)	1	2	3	4	
14	I feel numbness or tingling in my hands or feet (Numbness or tingling)	1	2	3	4	
15	I am troubled by stomach pain or indigestion (Stomach pain or indigestion)	1	2	3	4	
16	I often need to urinate (Frequent urination)	1	2	3	4	
17	My hands are usually dry and warm (Excessive sweating)	1	2	3	4	
18	My face feels flushed or hot (Facial flushing)	1	2	3	4	
19	I fall asleep easily and sleep well through the night (Sleep disturbance)	1	2	3	4	
20	I have nightmares	1	2	3	4	

Total Score: ________.

Note:

- Scoring uses a 1–4 scale. Items marked with * (5, 9, 13, 17, 19) are reverse-scored (4–1).
- Sum the scores of all 20 items to get the total score. Multiply the total score by 1.25, then round to the nearest integer to obtain the **standard score**.
- **Anxiety Evaluation Cutoff**:
    - Standard score ⩾ 50 indicates anxiety.

Date：|__|__|__|__|__|__|__|__|

    - 50–59: Mild anxiety
    - 60–69: Moderate anxiety
    - ⩾ 70: Severe anxiety
- Higher scores indicate a stronger tendency toward anxiety.

SF-36 Health Survey (Quality of Life Assessment)

1. General Health

Question	Options (Circle the most appropriate situation)
1. In general, your health is:	① Excellent ② Very good ③ Good ④ Fair ⑤ Poor
2. Compared to one year ago, how would you rate your health?	① Much better than one year ago ② Somewhat better than one year ago ③ About the same as one year ago ④ Somewhat worse than one year ago ⑤ Much worse than one year ago

3. Physical Functioning

The following questions relate to daily activities. Consider whether your health limits these activities and to what extent.

Activity	1 (Greatly limited)	2 (Somewhat limited)	3 (Not limited)
(1) Vigorous activities (e.g., running, lifting heavy objects, strenuous sports)	①	②	③
(2) Moderate activities (e.g., moving a table, sweeping, Tai Chi, simple exercises)	①	②	③
(3) Carrying groceries (e.g., shopping for vegetables)	①	②	③
(4) Climbing several flights of stairs	①	②	③
(5) Climbing one flight of stairs	①	②	③

Activity	1 (Greatly limited)	2 (Somewhat limited)	3 (Not limited)
(6) Bending, kneeling, or squatting	①	②	③
(7) Walking more than 1500 meters	①	②	③
(8) Walking 1000 meters	①	②	③
(9) Walking 100 meters	①	②	③
(10) Bathing or dressing yourself	①	②	③

4. Role Limitations Due to Physical Health

In the past 4 weeks, have you experienced the following problems with work or daily activities due to physical health?

Problem	1 (Yes)	2 (No)
(1) Cut down on the amount of time spent on work or other activities	①	②
(2) Accomplished less than you would like	①	②
(3) Were limited in the kind of work or activities	①	②
(4) Had difficulty performing work or other activities (e.g., required extra effort)	①	②

5. Role Limitations Due to Emotional Problems

In the past 4 weeks, have you experienced the following problems with work or daily activities due to emotional problems (e.g., feeling depressed or anxious)?

Problem	1 (Yes)	2 (No)
(1) Cut down on the amount of time spent on work or other activities	①	②
(2) Accomplished less than you would like	①	②
(3) Did work or activities less carefully than usual	①	②

6. Social Functioning

Question	Options
In the past 4 weeks, to what extent has your physical health or emotional problems interfered with your normal social activities with family, friends, neighbors, or groups?	① Not at all ② Slightly ③ Moderately ④ Quite a bit ⑤ Extremely

7. Bodily Pain

Question	Options
In the past 4 weeks, have you had bodily pain?	① None ② Very mild ③ Moderate ④ Severe ⑤ Very severe

8. Pain Interference

Question	Options
In the past 4 weeks, how much did pain interfere with your work or household chores?	① Not at all ② Slightly ③ Moderately ④ Quite a bit ⑤ Extremely

9. Mental Health

The following questions are about how you felt during the past 4 weeks. For each item, indicate how often you experienced it.

Feeling	6 (All of the time)	5 (Most of the time)	4 (A good bit of the time)	3 (Some of the time)	2 (A little of the time)	1 (None of the time)
(1) Felt full of life	①	②	③	④	⑤	⑥
(2) Been a very nervous person	①	②	③	④	⑤	⑥
(3) Felt so down that nothing could cheer you up	①	②	③	④	⑤	⑥
(4) Felt calm and peaceful	①	②	③	④	⑤	⑥
(5) Had a lot of energy	①	②	③	④	⑤	⑥

Date：|__|__|__|__|__|__|__|__|

Feeling	6 (All of the time)	5 (Most of the time)	4 (A good bit of the time)	3 (Some of the time)	2 (A little of the time)	1 (None of the time)
(6) Felt downhearted and blue	①	②	③	④	⑤	⑥
(7) Felt worn out	①	②	③	④	⑤	⑥
(8) Been a happy person	①	②	③	④	⑤	⑥
(9) Felt tired	①	②	③	④	⑤	⑥

10. Social Functioning (Additional)

Question	Options
Poor health interfered with social activities (e.g., visiting relatives or friends)	① All of the time ② Most of the time ③ A good bit of the time ④ Some of the time ⑤ A little of the time ⑥ None of the time

11. General Health Perceptions

For each statement, indicate which answer best describes your situation.

Statement	1 (Definitely true)	2 (Mostly true)	3 (Not sure)	4 (Mostly false)	5 (Definitely false)
(1) I seem to get sick more easily than others	①	②	③	④	⑤
(2) I am as healthy as people around me	①	②	③	④	⑤
(3) I expect my health to get worse	①	②	③	④	⑤
(4) My health is excellent	①	②	③	④	⑤

Total Score: _________.

Scoring Formula:

Final score = [(Actual score - Lowest possible score) / (Highest possible score - Lowest

possible score)] × 100.
Each domain is scored out of 100, with higher scores indicating better quality of life in that domain.

Ear Fullness Sensation Assessment

Has your sensation of ear fullness improved? Check (√) the appropriate option:

**Option**	**Check (√)**
No improvement in ear fullness	[ ]
Ear fullness improved but worsens with activity or fatigue	[ ]
Ear fullness significantly improved	[ ]
Ear fullness completely resolved	[ ]

Date：|__|__|__|__|__|__|__|__|

Field Details Patient Name Abbreviation __ __ __ __

Date Year: __ __ __ __ Month: __ __ Day: __ __

Research Completion Status Summary

Trial Conclusion

Adverse Events

**Adverse Event**	**Event 1**	**Event 2**	**Event 3**
**Adverse Event Description**			
**Start Date**	|__|__|__|__|__|__|__|__|	|__|__|__|__|__|__|__|__|	|__|__|__|__|__|__|__|__|
**End Date**	|__|__|__|__|__|__|__|__|	|__|__|__|__|__|__|__|__|	|__|__|__|__|__|__|__|__|
**Ongoing**	[ ] Yes [ ] No	[ ] Yes [ ] No	[ ] Yes [ ] No
**Severity**	[ ] Mild<br>[ ] Moderate<br>[ ] Severe	[ ] Mild<br>[ ] Moderate<br>[ ] Severe	[ ] Mild<br>[ ] Moderate<br>[ ] Severe
**Relatedness to Study Treatment**	[ ] Unrelated<br>[ ] Unlikely related<br>[ ] Possibly related<br>[ ] Related	[ ] Unrelated<br>[ ] Unlikely related<br>[ ] Possibly related<br>[ ] Related	[ ] Unrelated<br>[ ] Unlikely related<br>[ ] Possibly related<br>[ ] Related
**Relatedness to Non-Study Treatment**	Please describe: e.g., Possibly related to aspirin use	Please describe: e.g., Possibly related to aspirin use	Please describe: e.g., Possibly related to aspirin use
**Measures Taken for Study Treatment**	[ ] Increased treatment frequency<br>[ ] No change	[ ] Increased treatment frequency<br>[ ] No change	[ ] Increased treatment frequency<br>[ ] No change

Date：|__|__|__|__|__|__|__|__|

Adverse Event	Event 1	Event 2	Event 3
	[ ] Reduced treatment frequency<br>[ ] Not applicable<br>[ ] Unknown	[ ] Reduced treatment frequency<br>[ ] Not applicable<br>[ ] Unknown	[ ] Reduced treatment frequency<br>[ ] Not applicable<br>[ ] Unknown
**Other Measures Taken**	Please describe:	Please describe:	Please describe:
**Outcome**	[ ] Not resolved/relieved<br>[ ] Resolved/relieved<br>[ ] Resolving/relieving<br>[ ] Unknown	[ ] Not resolved/relieved<br>[ ] Resolved/relieved<br>[ ] Resolving/relieving<br>[ ] Unknown	[ ] Not resolved/relieved<br>[ ] Resolved/relieved<br>[ ] Resolving/relieving<br>[ ] Unknown
**Serious Adverse Event**	[ ] Yes [ ] No	[ ] Yes [ ] No	[ ] Yes [ ] No
**Led to Study Discontinuation**	[ ] Yes [ ] No	[ ] Yes [ ] No	[ ] Yes [ ] No

Research Completion Status Summary

Field	Details
**Subject's First/Last Treatment Date**	
**First Treatment Date**	|__|__|__|__|__|__|__|__| (YYYY/MM/DD)
**Last Treatment Date**	|__|__|__|__|__|__|__|__| (YYYY/MM/DD)
**Did the Subject Complete the Clinical Treatment Period?**	[ ] Yes [ ] No,<br>Discontinuation Date: |__|__|__|__|__|__|__|__| (YYYY/MM/DD)

Date：|_|_|_|_|_|_|_|_|

**Field**	**Details**
	[ ] Terminated by the principal investigator<br>[ ] Protocol violation (Any protocol deviation will be evaluated by the investigator and, after consultation with the principal investigator, determined if it is severe enough to warrant withdrawal), please specify: ______________
**Primary Reason for Trial Discontinuation (Select One)**	[ ] Adverse event, specify: ______________<br>[ ] No adverse event, but the investigator believes termination is in the subject's best interest for safety reasons<br>[ ] Lack of efficacy [ ] Subject withdrew informed consent<br>[ ] Lost to follow-up (including subject dropout)<br>[ ] Other reasons: ______________
**Did the Subject Complete the Follow-Up Period?**	[ ] Yes<br>[ ] No, Date of Last Contact: **///** (YYYY/MM/DD), Reason for Incomplete Follow-Up: ______________
**Primary Reason for Follow-Up Period Discontinuation (Select One)**	[ ] Terminated by the principal investigator<br>[ ] Protocol violation (Any protocol deviation will be evaluated by the investigator and, after consultation with the principal investigator, determined if it is severe enough to warrant withdrawal), please specify: ______________<br>[ ] Adverse event, specify: ______________<br>[ ] No adverse event, but the investigator believes termination is in the subject's best interest for safety reasons [ ] Lack of efficacy<br>[ ] Subject withdrew informed consent<br>[ ] Lost to follow-up (including subject dropout)<br>[ ] Other reasons: ______________

Date：|__|__|__|__|__|__|__|__|

Principal Investigator Declaration

I have reviewed all data recorded for each follow-up visit in the study medical records, and I confirm that these data are complete, accurate, and consistent with the source documents. All data recording was performed by me or individuals delegated by me, and we have signed the investigator signature list.

Field	Details
**Principal Investigator Signature**	__________
**Date**	|__|__|__|__||__|__||__|__|

Acupuncture Treatment Record Form

<table>
<tr><th>Treatment Session</th><th>Acupuncture Performed</th><th>Treatment Date (YYYY/MM/DD)</th><th>Remarks</th></tr>
<tr><td>First Acupuncture</td><td>[ ] √ [ ] ×</td><td>|____|__|__|</td><td></td></tr>
<tr><td>2nd Acupuncture</td><td>[ ] √ [ ] ×</td><td>|____|__|__|</td><td></td></tr>
<tr><td>3rd Acupuncture</td><td>[ ] √ [ ] ×</td><td>|____|__|__|</td><td></td></tr>
<tr><td>4th Acupuncture</td><td>[ ] √ [ ] ×</td><td>|____|__|__|</td><td></td></tr>
<tr><td>5th Acupuncture</td><td>[ ] √ [ ] ×</td><td>|____|__|__|</td><td></td></tr>
<tr><td>6th Acupuncture</td><td>[ ] √ [ ] ×</td><td>|____|__|__|</td><td></td></tr>
<tr><td>7th Acupuncture</td><td>[ ] √ [ ] ×</td><td>|____|__|__|</td><td></td></tr>
<tr><td>8th Acupuncture</td><td>[ ] √ [ ] ×</td><td>|____|__|__|</td><td></td></tr>
<tr><td>9th Acupuncture</td><td>[ ] √ [ ] ×</td><td>|____|__|__|</td><td></td></tr>
<tr><td>10th Acupuncture</td><td>[ ] √ [ ] ×</td><td>|____|__|__|</td><td></td></tr>
<tr><td>11th Acupuncture</td><td>[ ] √ [ ] ×</td><td>|____|__|__|</td><td></td></tr>
<tr><td>12th Acupuncture</td><td>[ ] √ [ ] ×</td><td>|____|__|__|</td><td></td></tr>
<tr><td>13th Acupuncture</td><td>[ ] √ [ ] ×</td><td>|____|__|__|</td><td></td></tr>
<tr><td>14th Acupuncture</td><td>[ ] √ [ ] ×</td><td>|____|__|__|</td><td></td></tr>
</table>

Note: Please mark √ or × in the "Acupuncture Performed" column. Do not leave it blank.

Investigator Signature: __________

Date：|_|_|_|_|_|_|_|_|
